# A systematic review and time-response meta-analysis of the optimal timing of elective caesarean sections for best maternal and neonatal health outcomes

**DOI:** 10.1186/s12884-020-03036-1

**Published:** 2020-07-08

**Authors:** Barbara Prediger, Tim Mathes, Stephanie Polus, Angelina Glatt, Stefanie Bühn, Sven Schiermeier, Edmund A. M. Neugebauer, Dawid Pieper

**Affiliations:** 1 Institute for Research in Operative Medicine, Witten/Herdecke University, Ostmerheimer Str. 200, 51109 Cologne, Germany; 2 Department of Obstetrics and Gynecology, Witten/Herdecke University, Marien Hospital Witten, Marienplatz 2, 58452 Witten, Germany; 3 Brandenburg Medical School - Theodor Fontane, Faculty of Health, Campus Neuruppin, Fehrbelliner Str.38, 16816 Neuruppin, Germany; 4 Interdisciplinary Centre for Health Services Research, Witten/Herdecke University, Alfred-Herrhausen-Straße 50, 58448 Witten, Germany

**Keywords:** Elective caesarean section, Term birth, Neonatal intensive care unit, Neonatal morbidity, Maternal morbidity, Gestational age, Time-response meta-analysis

## Abstract

**Background:**

The rate of caesarean sections (CS) has increased in the last decades to about 30% of births in high income countries. Many CSs are electively planned without an urgent medical reason for mother or child. An early CS though may harm the newborn. Our aim was to evaluate the gestational time point after the 37 + 0 week of gestation (WG) (after prematurity = term) of performing an elective CS with the lowest morbidity for mother and child by assessing the time course from 37 + 0 to 42+ 6 WG.

**Methods:**

We performed a systematic literature search in MEDLINE, EMBASE, CENTRAL and CINAHL in November 2018. We included studies that compared different time points of elective CS at term no matter the reason for elective CS. Our primary outcomes were the rate of admissions to the neonatal intensive care unit (NICU), neonatal death and maternal death in early versus late term elective CS. Various binary and dose response random effects meta-analyses were performed.

**Results:**

We identified 35 studies including 982,749 women. Except one randomised controlled trial, all studies were cohort studies. We performed a linear time-response meta-analysis on the primary outcome NICU admission on 14 studies resulting in a decrease of the relative risk (RR) to 0.63 (95% CI 0.56, 0.71) from 37 + 0 to 39 + 6 WG. RR for neonatal death showed a decrease to 39 + (0–6) WG (RR 0.59 95% CI 0.43 to 0.83) and increase from then on (RR 2.09 95% CI 1.18 to 3.70) assuming a U-shape course and using a cubic spline model for meta-analysis of four studies. We only identified one study analyzing maternal death resulting in RR of 0.38 (95% CI 0.04 to 3.40) for 37 + 0 + 38 + 6 WG versus ≥39 + 0 WG.

**Conclusion:**

Our systematic review showed that elective CS (primary and repeated) before the 39 + 0 WG lead to more NICU admissions and neonatal deaths, although death is rare and increases again after 39 + 6 WG. We did not find enough evidence on maternal outcomes. There is a need for more research, considering maternal outcomes to provide a balanced decision between neonatal and maternal health.

**Systematic review registration:**

Registered in PROSPERO (CRD42017078231).

## Background

While the World Health Organization (WHO) states that there is no medical reason for a higher rate of CSs than 10–15%, the rates of Caesarean Section (CS) in high income countries have increased to about 30% of all births in the last decades [1–3]. It is assumed that a high number of CSs is electively planned without an urgent medical need neither in women nor the unborn. A previous CS is the most common reason for performing an elective CS. Researchers from the UK and USA showed that only 50% of women in the UK undergo vaginal birth after CS (VBAC) while there are with only 10% even less in the USA, even though it is recommended for the majority of women with prior CS [4, 5]. Withal there is no unanimity when the optimal time point of performing an elective CS could be. While 97% of elective CSs are performed beyond 37 + 0 WG, about 60% of elective CSs are performed in, or beyond 39 (39 + 0 to 39 + 6) WG, according to an analysis of 63 English NHS trusts [6].

The reason behind is that women with a scarred uterus may have diverse risks in following pregnancies and placentation abnormalities may occur more often. The risk of scar rupture may increase with the growing unborn in the last weeks of pregnancy [7]. Injuries to the bladder and a higher risk of bleeding needing transfusion is assumed. And because of this even a higher mortality rate might be connected to late term elective caesareans compared to early term caesareans before the beginning of labor [8]. Women without prior CS/intact uterus are not touched by those risks. Still labor can occur before the planned time point of CS which may result in an emergency CS which is connected with higher risks [9].

But in childbirth the risks for the neonate may not go along with those for the mother and is even though at term (37 + 0 WG) under various health risk. Lungs are mature in 37 + 0 WG, but neonates born by CS have a general higher risk of respiratory disorders. This is especially linked to early term CS [10].

The two guidelines “Caesarean Section” by NICE and “Birth after previous caesarean birth” by the Royal College for Obstetricians & Gynecologists examine if early term CS increases respiratory morbidity of the neonate. Both recommend to perform elective CS not before the 39 + 0 WG [11, 12]. Furthermore the American College of Obstetricians and Gynecologists recommend in their committee opinions 764 and 765 to not perform any indicated deliveries (both induction of labor and caesarean section) before the 39 + 0 WG, except for some specific pregnancy complications or comorbidities [13, 14]. In uncomplicated dichorionic diamniotic twin pregnancies, elective delivery (vaginal or by CS) should be offered in 37 + (0–6) WG according to the guideline “Twin and triplet pregnancy” from NICE. Risks are increasing from 38 + 0 WG onwards. Nevertheless, about 60% of neonates, are born spontaneously preterm – before 37 + 0 WG [15]. This fact may result in a relevant number of elective CS performed late preterm.

But high level evidence is lacking. There are currently no meta-analyses available which sum up the existing evidence.

As there is an ongoing trend towards more electively planned CSs, it is essential to provide a time point for the CS with the lowest risk for both, mother and child, comparing early term (37 + 0 to 38 + 6 WG) and late term (≥39 + 0 WG) delivery.

We performed a systematic review of the literature to evaluate the optimal time point with

- low risk of mortality and morbidity for mothers
- low risk for the neonate for mortality and morbidity

Beforehand, in 2016, we performed a systematic review on behalf of the German Federal Ministry of Health to answer the present question [16]. Herewith we updated this review and also aimed to expand the reach of the findings with this update in English. Moreover in the original review we performed a random-effects meta-analysis only comparing 37 + 0 to 38 + 6 WG with ≥39 + 0 WG, in this update we performed another type of meta-analysis showing a linear time-response relationship.

## Methods

### Protocol and registration

We registered our review at PROSPERO (CRD42017078231) and published the protocol [17].

### Eligibility criteria

We included women with a planned CS at term (≥37 + 0 WG), regardless if it was first caesarean or repeated CS. We included studies with singleton and multiple pregnancies. Even though multiple pregnancies deviate much from singleton pregnancies we assumed similar uncertainties about the timing of elective CS. Our interest were planned CSs at various time points. The primary outcomes were neonatal death, NICU admission and maternal death. Secondary outcomes are for neonates: hospitalization ≥5 days, respiratory morbidity, respiratory distress syndrome (RDS), transient tachypnea of the neonate (TTN), pneumothorax, hypoglycemia (Depending on the age at assessment: 0–3 h: < 2.0 mmol/l; 3–24 h < 2.2 mmol/l; > 24 h < 2.5 mmol/l) [18], Apgar Score < 7, hyperbilirubinemia needing phototherapy (jaundice), near miss (a newborn infant who nearly died but who survived a complication occurring during pregnancy, childbirth, or in the first 7 days after the termination of pregnancy). For mothers we included following outcomes: hysterectomy, bleeding needing transfusion, and near miss miss (a woman who nearly died but survived a complication that occurred during pregnancy, childbirth or within 42 days of termination of pregnancy). We report outcomes with unspecific definition like respiratory morbidity as it is defined in the relevant study. The inclusion was limited to studies in WHO Stratum A. This covers states with very low child and very low adult mortality including western Europe, North-America and various Western-Pacific states [19]. We chose this stratum because of the very low general (and child) mortality and comparable access to health services, but also because of comparable CS rates and similar indications for CS, such as organizational reasons on hospital, personal maternal and clinical base [20]. We did not define any other exclusion criteria regarding the population. We considered randomized controlled trials (RCTs), quasi RCTs and cohort studies. RCTs are much more difficult to conduct (E.g. due to spontaneous onset of labor) and we expected low numbers of RCTs. Even though cohort studies are suspected to have higher risks of systematic biases, we assumed a high amount of data owing to birth registries. We did not make any restrictions regarding the language and publication date.

### Information sources

We searched MEDLINE, EMBASE, CENTRAL and CINAHL on 29th of November 2018. We did not restrict the search to any language or publication date. Study registries were searched for new and unpublished studies (ClinicalTrials, Deutsches Register Klinischer Studien and EU clinical trials register). To identify grey literature we searched Google Scholar additionally.

We also checked the references of included studies, guidelines and systematic reviews and if necessary contacted authors for additional data.

### Search strategy

The search strategy was developed using MeSH terms and text words and a librarian checked the strategy by applying the PRESS checklist [21]. The search strategies are available in Additional file 1.

### Study selection

Records identified through the searches were added to an Endnote X7 database and duplicates were removed. Two reviewers independently assessed the relevance of the identified titles and abstracts according to the inclusion criteria. Studies which were included for full text review again were independently assessed by the same two reviewers. Differences were discussed until a consensus was found or a third reviewer was included.

### Data collection

Data was collected in an a priori-piloted abstraction table by one reviewer, the other reviewer monitored all entries for completeness and accuracy. We extracted data directly in an excel sheet. If the study authors only reported adjusted effect measures in their publications we raised enquiries to the authors for unadjusted data.

### Data items

We extracted following study characteristics: Author, publication year, region, setting, study design, recruitment period, exclusion criteria, patient characteristics (Age, body-mass index, ethnicity, diseases, parity, prior CS, indication for CS, marital/educational/socioeconomical status, payer, smoking status), time points measured, outcomes. All outcomes are collected as dichotomous variables and for each time point.

### Risk of bias assessment

Two reviewers independently assessed risk of bias. We discussed differences until we found a consensus or a third reviewer was included. For RCTs we used the Cochrane Risk of Bias Tool [22]. For cohort studies we used the ROBINS-I Tool [23]. We first assessed risk of bias on study level and summarized it on outcome level.

### Data synthesis

We only pooled studies that were assessed to be sufficient clinical homogenous judged by reviewers with clinical expertise. If studies were sufficiently clinically homogenous, a random-effects meta-analysis was performed. We performed a multivariate dose-response meta-analysis for pooling outcomes where time starting with 37 + 0 WG up to 42 + 6 WG in weekly steps represented the different doses. We examined visually for each outcome if the assumed time-response relationship was effectively present and how the relationship was shaped [24]. Therefore, we created plots showing the intervention effect for each study over time. Based on these curves we determined the shape (e.g. linear, U-shape) specified in the dose-response meta-analysis. For most neonatal (adverse) outcomes we recognized a regressive or u-shape (with a minimum at week 39) and for maternal (adverse) outcomes a progressive trend [10, 16, 25]. In the first stage of our analysis, we estimated a time-response curve (i.e. gestational week-outcome) for each study across WG values observed in the whole dataset. In the second stage these curves were pooled into an overall gestational week-outcome curve. The time-response analysis followed the two-stage method for dose-response-meta-analysis by Greenland & Longnecker [26]. We calculated study-specific slopes (linear trends) and 95% confidence intervals from the natural logs of the reported effect measures and confidence intervals across WG taking the correlations between RRs into account. In case of the reference category being not the lowest category we first recalculated the data in such a way that (depending on the shape) week 39 or the lowest category was the reference category. In cases where this was not possible, we excluded the categories below the reference category for the linear time-response analysis. For studies reporting ranges of weeks the midpoint of the lower and upper cut-off was assigned for each category. When upper and lower categories were open ended, the lower and upper cut-off value was 37 and 42 weeks. Again the midpoint of the lower and upper cut-off was assigned for each category. When authors reported the median or mean per category this was used to assign the corresponding RR for each study.

Statistical heterogeneity was assessed with the Q test, I^2^ statistic and prediction intervals. Prediction intervals can help with the interpretation of heterogeneity, by presenting the expected range of true treatment effects in similar research [27, 28].

All analyses were performed with R 3.3.2 using the meta and dosresmeta packages [29, 30].

If data were too heterogeneous, we performed a structured narrative analysis of the outcome. We used GRADE to rate the certainty in evidence [31]. Two reviewers independently performed the GRADE assessment for each outcome with the GRADEpro GDT Software. Domains assessed with the GRADE approach are risk of bias, inconsistency, indirectness, imprecision, publication bias, large effects, confounding and dose response gradients.

### Risk of bias across studies

Publication bias: We assessed publication bias by visual inspection of the funnel plot. We assumed publication bias if we found asymmetry in the plots. Furthermore, we applied Egger’s test and Begg’s test [32, 33]. A *p*-value < 0.1 was considered statistically significant.

Selective reporting within studies: If available, study protocols were checked and compared with reporting in studies. We searched clinicaltrials.gov to detect protocols if not stated otherwise. We desisted from contacting authors of the publications of registries for protocols.

### Additional analyses

We performed subgroup analyses for repeat CS vs. first CS and for studies including exclusively multiple pregnancies. Besides general deviations in multiple pregnancies compared to singleton pregnancies we assumed that CS is planned earlier than 37 + 0 WG to 42 + 6 WG more often, and may need a time-response analysis considering other comparisons of WG.

In a sensitivity analysis for primary outcomes, we conducted a univariate random effects meta-analysis (37 + 0 to 38 + 6 vs ≥39 + 0 WG) to demonstrate reliability of the results. We used the Paule and Mandel heterogeneity variance estimator and modified Hartung-Knapp confidence intervals for the pooled estimates [34, 35].

## Results

### Study selection

**Fig. 1:**
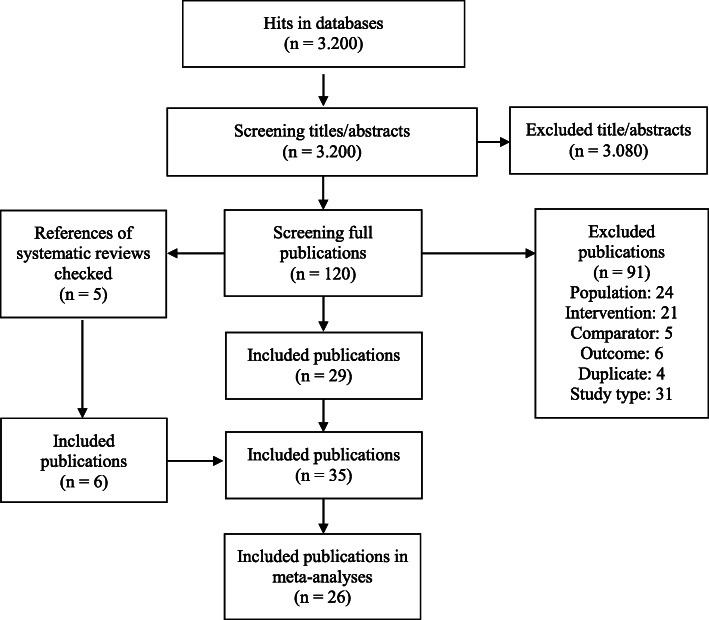
Flow-diagram of study selection

We identified 3200 hits in the databases after duplicate removal. One hundred twenty publications were screened in full text of which we included 29 in the review. Moreover we identified six references by screening the reference lists of five systematic reviews. The references from the guidelines, the search in Google Scholar and the search in registries resulted in no additional inclusions. The included and excluded (with reason) studies are presented in Additional file 1 and Fig. 1.

### Study characteristics

Of the 35 included publications, three, Brookfield, Chiossi and Tita et al. used the same birth registry [36–38]. Also Vilchez et al. and Zanardo et al. published two papers from the same cohort [39, 40]. We used the first publications and added outcome data from the following publications. Except for one RCT from Glavind et al. all studies were cohort studies [25]. One study, Wilmink et al. examined only twin births. Two studies from Japan, Nakashima et al. and Yamazaki et al., and one from Germany, Gawlik et al., only compared the 37 + (0–6) to the 38 + (0–6) WG and four, Doan, McAlister, Nir and Zanardo et al., did not report the single WG but compared 37 + (0–6) + 38 + (0–6) to ≥39 + (0–6) week [41–49]. These eight studies could not be included in any meta-analysis. Patient numbers of the included studies ranged from 96 to 785,340 with a median of 13,888. Twenty-two studies reported the exclusion of women with multiple pregnancies and 15 studies the exclusion of pregnancies with fetal congenital anomalies. In 24 studies exclusion criteria for mothers with any morbidity influencing the timing of birth (e.g. hypertension, diabetes, placenta previa) were reported. Nineteen Studies evaluated NICU admission and six studies evaluated neonatal death. Maternal death was only assessed in one study. None of the studies reported or considered near miss for neonates or mothers. One study, Terada et al., reported outcomes exclusively on oxygen supplementation and respiratory support with overlapping patients, so we did not include this in the meta-analysis [50]. For detailed study and patient characteristics see Additional file 1 and Table 1.

**Table 1:** Characteristics of included studies

Study	Study type	Setting	Recruiting period	n	Week of gestation	≥1 C-Section
Alderdice et al. 2005 [51]	Cohort study	Northern Ireland, multicentric	2001–2002	2553	37, 38, 39, 40, 41	No
Bailit et al. 2010 [52]	Cohort study	USA, multicentric	2002–2008	3959	34, 35, 36, 37, 38, 39, 40, 41, 42	No
Balchin et al. 2008 [53]	Cohort study	England, multicentric	1988–2000	20,891	37, 38, 39, 40	No
Brookfield et al. 2017 [36]	Cohort study	USA, multicentric, see Tita 2009, Chiossi 2013	1999–2002	15,602	37, 38, 39, 40, ≥41	Yes
Chiossi et al. 2013 [37]	Cohort study	USA, multicentric, see Tita 2009, Brookfield 2017	1999–2002	14,865	37, 38, 39, 40, 41	Yes
Clark et al. 2009 [54]	Cohort study	USA, multicentric	2007	1851	37, 38, ≥39	Both
Doan et al. 2014 [44]	Cohort study	Australia,1 center	1998–2009	14,447	37–38, 39–41	No
Farchi 2010	Cohort study	Italy, multicentric	2003–2005	13,329	37, 38, 39, 40–41	Yes
Finn et al. 2016 [55]	Cohort study	Ireland, 1 center	2008–2012	4242	37, 38, 39, 40, 41	No
Gawlik et al. 2015 [43]	Cohort study	Germany, 1 center	2006–2011	503	37, 38–40	Yes
Glavind 2013	RCT	Denmark, multicentric	2009–2011	1274	38, 39	No
Graziosi et al. 1998 [56]	Cohort study	Netherlands, 1 center	1990–1995	272	37, 38, 39, 40, 41	No
Hansen et al. 2008 [57]	Cohort study	Denmark, 1 center	1998–2006	2687	37, 38, 39, 40, 41	No
Many et al. 2006 [58]	Cohort study	Israel, −	–	278	38, 39, 40, 41	No
Matsuo et al. 2008 [59]	Cohort study	Japan, 1 center	1994–2005	364	37, 38, ≥39	No
McAlister et al. 2013 [45]	Cohort study	USA, multicentric	2008–2009	4125	37–38, 39–41	No
Melamed et al. 2014 [60]	Cohort study	Israel, 1 center	2010–2011	377	38, 39	≥2
Morrison et al. 1995 [61]	Cohort study	England, 1 center	1985–1993	2341	37, 38, 39, 40, ≥41	No
Nakashima et al. 2014 [41]	Cohort study	Japan, 1 center	2006–2012	684	37, 38	No
Nir et al. 2012 [46]	Cohort study	Israel, 1 center	2007–2009	1050	37–38, ≥39	No
Parikh et al. 2014 [62]	Cohort study	USA, multicentric	2008–2011	14,613	37, 38, ≥39	No
Resende 2014	Cohort study	Portugal, 1 center	2003–2013	3123	37, 38, 39, 40, 41	No
Terada et al. 2014 [50]	Cohort study	Japan, 1 center	2006–2013	1936	37, 38, 39–40, 41	No
Tita et al. 2009 [38]	Cohort study	USA, multicentric, see Chiossi 2013, Brookfield 2017	1996–2006	13,258	37, 38, 39, 40, 41, 42	Yes
Tracy et al. 2007 [63]	Cohort study	Australia, multicentric	1999–2002	43,059	37, 38, 39, 40, 41	No
Van den Berg et al. 2001 [64]	Cohort study	Netherlands, 1 center	1994–1998	324	37, 38, ≥39	No
Vidic 2016	Cohort study	Slovenia, multicentric	2002–2012	7364	37, 38, 39, 40, ≥41	No
Vilchez et al. 2014 [39]	Cohort study	USA, multicentric, see Vilchez 2015	2004–2008	785,340	37, 38, 39, 40, 41	Yes
Vilchez et al. 2015 [40]	Cohort study	USA, multicentric, see Vilchez 2014	2004–2008	483,052	37, 38, 39, 40, 41	Yes
Wilmink et al. 2010 [65]	Cohort study	Netherlands, multicentric	2000–2006	20,973	37, 38, 39, 40, 41, 42	No
Wilmink et al. 2012 [66]	Cohort study	Netherlands, multicentric, twins	2000–2007	4557	35, 36, 37, ≥38	No
Yamazaki et al. 2003 [42]	Cohort study	Japan, 1 center	1998–2000	96	37, 38	No
Zanardo et al. 2004, two publications [48, 49]	Cohort study	Italy, 1 center	1998–2000	1284	37–38,39–41	No
Zanardo et al. 2007 [67]	Cohort study	Italy, multicentric	2002–2003	9988	37, 38, 39, 40–41 + 6	No

≥1 C-Section refers to studies including women who had at least one caesarean section before

### Risk of bias within studies

**Fig. 2:**
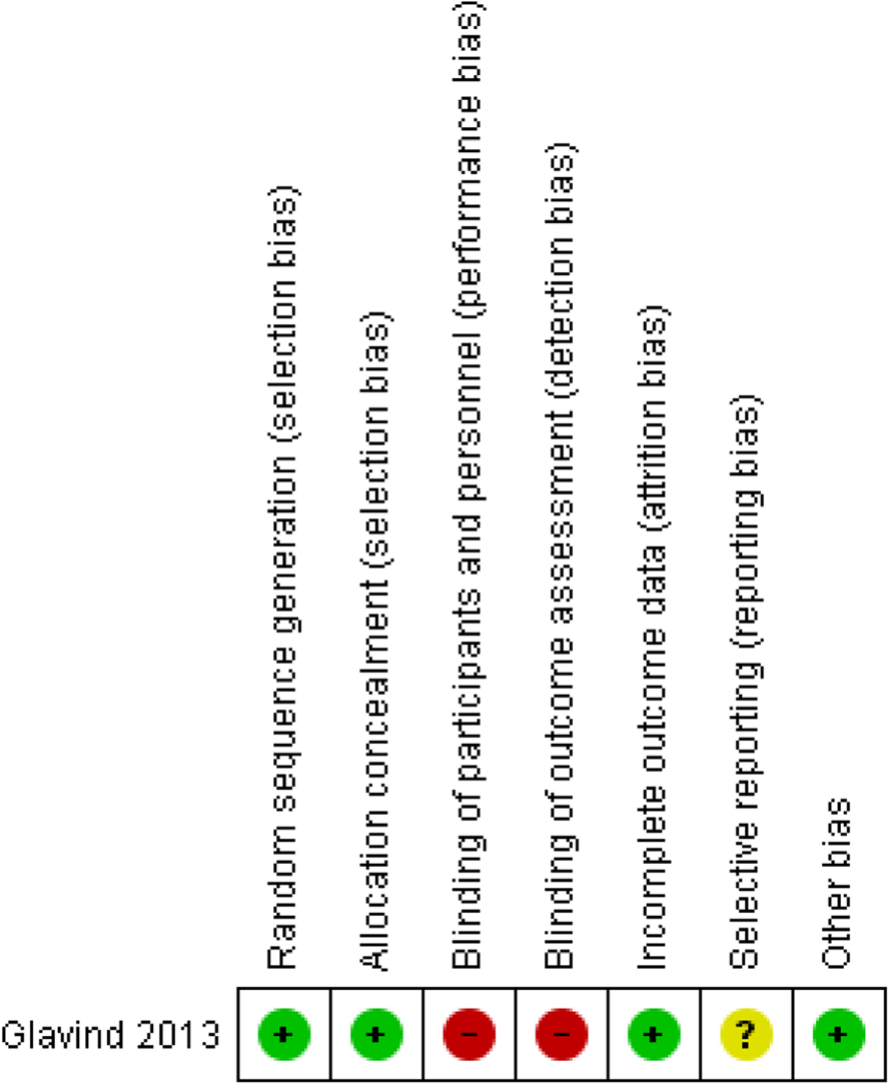
Risk of Bias assessment in RCTs for NICU admission

Risk of bias was assessed with the Cochrane Risk of Bias tool in the RCT from Glavind et al. see Fig. 2. We assumed a moderate overall risk of bias for the study of Glavind et al. attributable to the missing blinding. All other studies were assessed with the ROBINS-I tool. Consistently throughout all studies confounding and selection of participants were the main issues and we assumed at least serious risk of bias in these domains, see Table 2. The detailed ratings to each bias domain can be found in Additional file 1.

**Table 2:** Risk of bias assessment in cohort studies

Study	Outcome	Bias due to confounding	Bias in selection of participants into the study	Bias in classification of interventions	Bias due to deviations from intended interventions	Bias due to missing data	Bias in measurement of outcomes	Bias in selection of the reported result	Overall bias
Alderdice et al. 2005 [51]	NICU	S	C	L	L	NI	S	L	C
	Respiratory outcomes	S	C	L	L	NI	M	L	C
Bailit et al. 2010 [52]	NICU	S	S	L	L	NI	S	L	S
	Sepsis	S	S	L	L	NI	M	L	S
	(M) Hysterectomy	S	S	L	L	NI	L	L	S
Balchin et al. 2008 [53]	Respiratory outcomes	S	C	L	L	NI	M	L	C
Brookfield et al. 2017 [36]	Respiratory outcomes	S	S	L	L	NI	M	L	S
Chiossi et al. 2013 [37]	NICU, Apgar score	S	S	L	L	NI	S	L	S
	Death	S	S	L	L	NI	L	L	S
	Respiratory outcomes, sepsis	S	S	L	L	NI	M	L	S
	(M) Death, hysterectomy	S	S	L	L	NI	L	L	S
	(M) Bleeding	S	S	L	L	NI	S	L	S
Clark et al. 2009 [54]	NICU	S	S	L	L	NI	S	L	S
Doan et al. 2014 [44]	NICU, Apgar Score, jaundice	S	S	L	L	NI	S	L	S
	Death, hypoglycemia	S	S	L	L	NI	L	L	S
	Respiratory outcomes	S	S	L	L	NI	M	L	S
Farchi 2010	Respiratory outcomes	S	S	L	L	NI	M	L	S
Finn et al. 2016 [55]	NICU	S	S	L	L	NI	S	L	S
	Respiratory outcomes	S	S	L	L	NI	M	L	S
Gawlik et al. 2015 [43]	NICU, Apgar score	S	S	L	L	NI	S	L	S
Graziosi et al. 1998 [56]	NICU, jaundice	S	S	L	L	NI	S	L	S
	Respiratory outcomes	S	S	L	L	NI	M	L	S
Hansen et al. 2008 [57]	Respiratory outcomes	S	S	L	L	NI	M	L	S
Many et al. 2006 [58]	Respiratory outcomes	S	C	L	L	NI	M	L	C
Matsuo et al. 2008 [59]	Respiratory outcomes	S	C	L	L	NI	M	L	C
McAlister et al. 2013 [45]	NICU	S	C	L	L	NI	S	L	C
Melamed et al. 2014 [60]	NICU, Apgar score, jaundice	S	S	L	L	NI	S	L	S
	Death, hypoglycemia	S	S	L	L	NI	L	L	S
	Respiratory outcomes	S	S	L	L	NI	M	L	S
	(M) Hysterectomy	S	S	L	L	NI	L	L	S
	(M) bleeding	S	S	L	L	NI	S	L	S
Morrison et al. 1995 [61]	Respiratory outcomes	S	C	L	L	NI	M	L	C
Nakashima et al. 2014 [41]	NICU, jaundice	S	S	L	L	NI	S	L	S
	Respiratory outcomes	S	S	L	L	NI	M	L	S
	Hypoglycemia, sepsis	S	S	L	L	NI	L	L	S
Nir et al. 2012 [46]	NICU, Apgar score, jaundice	S	S	L	L	NI	S	L	S
	Respiratory outcomes	S	S	L	L	NI	M	L	S
	Hypoglycemia	S	S	L	L	NI	L	L	S
Parikh et al. 2014 [62]	NICU	S	S	L	L	NI	S	L	S
	Death	S	S	L	L	NI	L	L	S
Resende 2015	NICU	S	S	L	L	NI	S	L	S
	Respiratory outcomes	S	S	L	L	NI	M	L	S
	Hypoglycemia	S	S	L	L	NI	L	L	S
Terada et al. 2014 [50]	Respiratory outcomes	S	S	L	L	NI	M	L	S
Tita et al. 2009 [38]	NICU	S	S	L	L	NI	S	L	S
	Respiratory outcomes	S	S	L	L	NI	M	L	S
	Hypoglycemia, sepsis	S	S	L	L	NI	L	L	S
Tracy et al. 2007 [63]	NICU	S	S	L	L	NI	S	L	S
Van d. Berg et al. 2001 [64]	NICU	S	C	L	L	NI	S	L	C
	Respiratory outcomes	S	C	L	L	NI	M	L	C
Vidic 2016	NICU, Apgar score, jaundice	S	S	L	L	NI	S	L	S
	Respiratory outcomes	S	S	L	L	NI	M	L	S
	Hypoglycemia	S	S	L	L	NI	L	L	S
Vilchez et al. 2014 [39]	NICU, Apgar score	S	S	L	L	NI	S	L	S
Vilchez et al. 2015 [40]	Death	S	S	L	L	NI	L	L	S
Wilmink et al. 2010 [65]	NICU, Apgar score, jaundice	S	S	L	L	NI	S	L	S
	Death, hypoglycemia, sepsis	S	S	L	L	NI	L	L	S
	Respiratory outcomes	S	S	L	L	NI	M	L	S
Wilmink et al. 2012 [66]	NICU, Apgar score	S	S	L	L	NI	S	L	S
	Death, hypoglycemia, sepsis	S	S	L	L	NI	L	L	S
	Respiratory outcomes	S	S	L	L	NI	M	L	S
Yamazaki et al. 2003 [42]	Respiratory outcomes	S	S	L	L	NI	M	L	S
	Hypoglycemia	S	S	L	L	NI	L	L	S
Zanardo et al. 2004, two publications [48, 49]	Respiratory outcomes	S	S	L	L	NI	M	L	S
Zanardo et al. 2007 [67]	Respiratory outcomes	S	S	L	L	NI	M	L	S

Risk of bias assessment according to ROBINS-I tool. The seven bias domains are individually assessed for each study. The evaluation options are: *L* Low; *M* Moderate; *S* Serious; *C* Critical; *NI* No Information. Respiratory outcomes include all respiratory outcomes measured. Outcomes were summarized according to their risk of bias assessment

(M): Maternal outcomes

A number of studies attempted to control confounding by multivariable logistic regression but we could not use these data for the meta-analyses because the regarded adjustment factors varied widely. Because we pooled and mainly reported the univariate analysis, risk of confounding was assessed for this analysis. Frequent confounders were maternal age, ethnicity, maternal and neonatal comorbidities, methods to determine gestational age and study setting. Women, who were planned to have elective CS in later term ≥39 + (0–6) WG but needed unplanned CS before term because of complications, are at higher risk for drop out, so the number of healthy women with uncomplicated pregnancies potentially rises in late term CS. In contrast, women who are suspected to have more complications during birth are terminated to an earlier CS, which leads to increasing numbers of complicated pregnancies in early term CS. Therefore, we rated almost all studies as critical or serious risk of bias.

We could not see any risk of bias regarding the classification nor deviation from the intended intervention. We could not determine if there was a risk of bias because of missing data, as none of the studies described how missing data was dealt with, nor if there was missing data. Risk of bias in measurement of outcomes was driven by the suspected influence of the knowledge about the timing of CS on outcome measures. The outcome measure for death or hysterectomy is not influenced by the knowledge of term (objective outcome) whereas the neonatologists/obstetricians judgement about NICU admission is highly influenced (subjective outcome). We did not find an indication for selective reporting of the results in any study. Table 2 shows the risk of bias assessment on study and outcome level.

### Risk of bias across studies

**Fig. 3:**
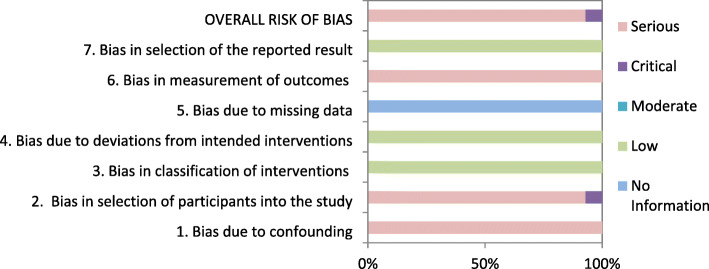
Risk of Bias assessment for NICU admission

The overall body of evidence assessment resulted in an assumption of serious or critical risk of bias. Figure 2 shows the risk of bias assessment for the outcome NICU admission. We did not produce graphs for each outcome as there would be nearly no difference in the graphs (Fig. 3).

All meta-analyses except the one for NICU admission included less than ten studies. We were only able to evaluate publication bias for NICU admission, which we did by consulting the funnel plot, which did not suggest publication bias (see Additional file 1). Both, Eggers and Beggs test did not indicate publication bias (Eggers test: *p*-value: 0.46; Beggs test: *p*-value: 0.83).

### Results of individual studies

Individual study results for NICU admission, neonatal and maternal death can be found in Additional file 1. We only identified one study from Chiossi et al., which analyzed maternal mortality [37]. The cases are very rare (1 in week 38, 4 in week 39) and we calculated a RR of 0.38 (95% CI 0.04 to 3.40, very low quality of evidence) for 37 + 0 to 38 + 6 WG versus ≥39 + 0 WG.

We identified 8 studies which we could not include in any meta-analysis for various reasons. Doan et al., Gawlik et al., McAlister et al., Nir et al., and Zanardo et al. reported outcomes for 37 + (0–6) + 38 + (0–6) WG versus ≥39 + 0 WG and not for individual weeks [44–46, 48, 49]. There were two studies from Japan and one from Germany that compared 37 + (0–6) WG to 38 + (0–6) [41] [43]. [42] They all showed similar results like the other studies; less NICU admission in the later WG.

### Synthesis of results

**Fig. 4:**
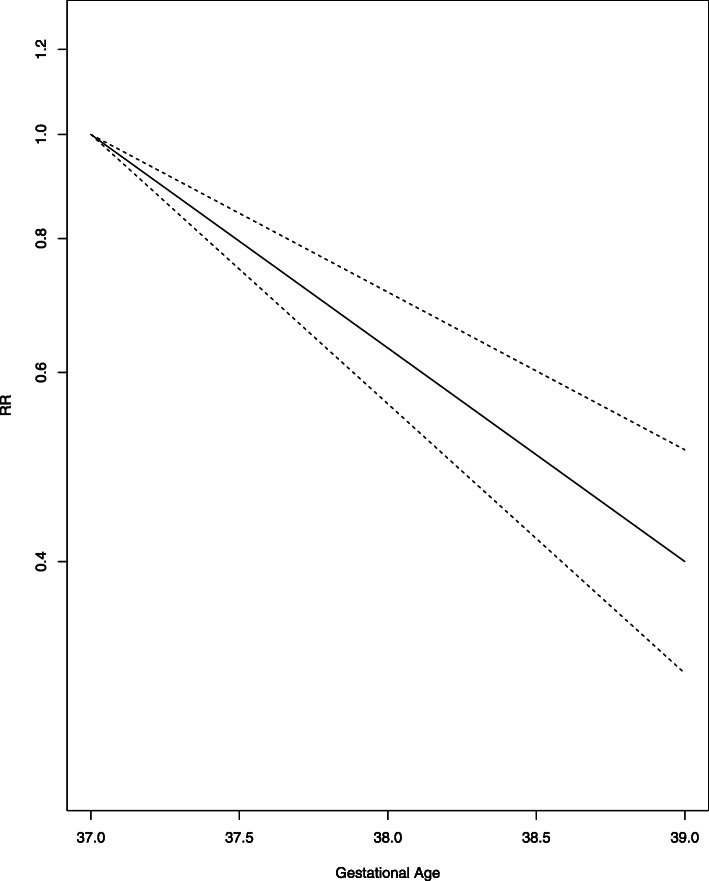
NICU admission

**Fig. 5:**
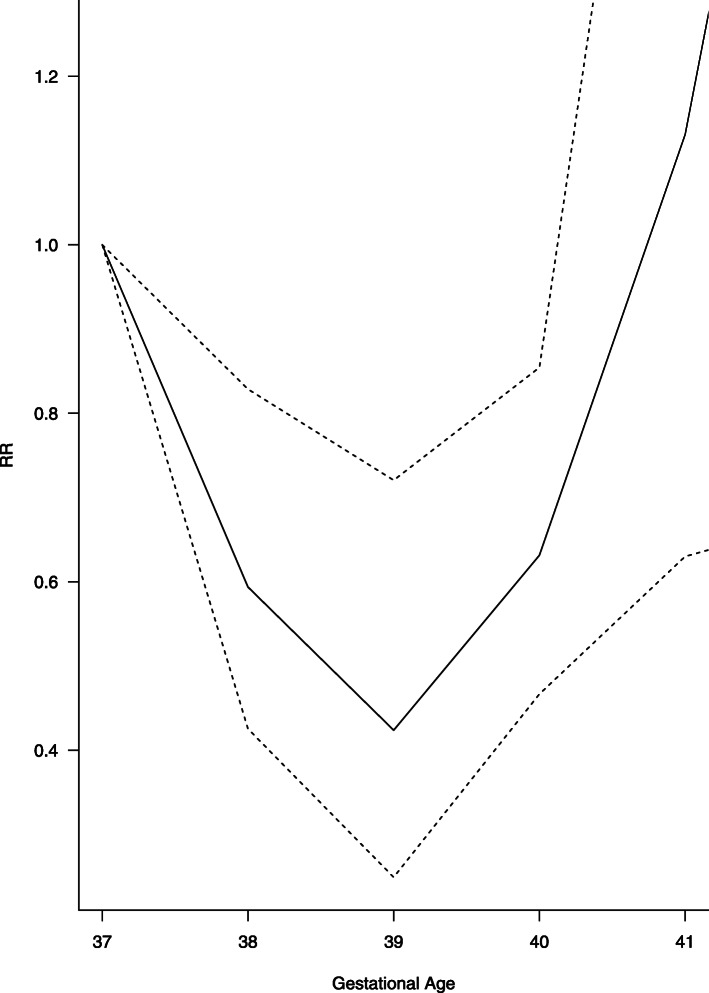
Neonatal death

We extracted the outcome data for each WG study wise in Excel. We calculated RRs with the reference category 39 + (0–6) WG and created graphs presenting the RRs over time. For each outcome and for each study, graphs were produced in the same manner and we visually inspected if a linear trend could be expected. Figures 4 and 5 show the graphs presenting the development of the primary outcomes NICU admission and neonatal death over time. The curves show the RR of the pooled 14 studies on NICU admission and respectively 4 studies on neonatal death. Both graphs are accompanied by the upper and lower CI. The course of NICU admission is decreasing from 37 + 0 to 39 + 6 WG, while the course of neonatal death shows the u-shape from 37 + 0 to 42 + 6 WG with the lowest at 39 + 0–6 WG. See Additional file 1 for the illustration of individual study results, which are underlying the models chosen.

We performed linear time-response meta-analyses for the outcomes NICU admission, respiratory morbidity, hypoglycemia, Apgar score < 7, jaundice, RDS, TTN, pneumothorax, maternal hysterectomy and maternal blood transfusions. The RR for NICU admission was 0.63 (95% CI 0.56 to 0.71, I^2^ = 95.4% low quality of evidence) (See Fig. 4) for each additional WG. All outcomes except Apgar score < 7, pneumothorax and both maternal outcomes showed a significant higher risk ratio the earlier the CS was performed. Except for sepsis, hypoglycemia, maternal hysterectomy and blood transfusion, all analyses showed high heterogeneity with I^2^ > 30%. See Table 3 for the individual results of the meta-analyses. All studies had a serious or critical risk of bias and therefore we rated the certainty of evidence according as low or very low, see Table 4. Only hypoglycemia was assessed as moderate certainty of evidence. Three other meta-analyses were cubic spline time-response meta-analyses with 39 + (0–6) WG as the reference. Incidence for neonatal death, sepsis and hospitalization ≥5 days all showed U-shaped curves with a minimum at 39 + (0–6) WG, i.e. a decreasing incidence form the 37 + 0 WG to the 39 + (0–6) WG and rising incidence from the 40 + 0 WG. The RR for neonatal death from 37 + 0 to 39 + 6 WG drops to 0.59 (95% CI 0.43 to 0.83, I^2^ = 77.5% low quality of evidence) and after 39 + 6 rises to 2.09 (95% CI 1.18 to 3.70, I^2^ = 77.5% low quality of evidence) (see Fig. 5). Sepsis and hospitalization show similar significant effects (see Table 3). The display of the GRADE evaluation in Table 4 is insufficient for the reporting the results of the cubic spline model. Therefore we chose to report the results as free text.

**Table 3:** Results of primary and subgroup meta-analyses by outcome

Meta-analyses	Studies	References	Patients n	Shape of association	Risk ratio	95% CI	I^2^
NICU admission all	14	[25, 37, 39, 51, 52, 54–56, 60, 62, 63, 65, 68, 69]	898,272	Linear dose-response^a^	0.63	0.56–0.71	95.4
Neonatal death	4	[37, 40, 62, 65]	533,880	U-Shape^b^	< 39: 0.59 ≥39: 2.09	0.43–0.83 1.18–3.70	77.5
Respiratory morbidity	9	[51, 53, 55, 57, 58, 61, 64, 65, 68]	57,693	Linear dose-response^a^	0.64	0.51–0.79	95.2
Hospitalization ≥5 days	5	[38, 62, 65, 68, 69]	59,331	U-Shape^b^	< 39 0.52 ≥39 2.00	0.36–0.75 1.40–2.86	96.2
Sepsis	4	[37, 52, 65, 68]	42,381	U-Shape^b^	< 39 0.55 ≥39 3.57	0.44–0.67 1.87–6.78	21.8
Hypoglycemia	6	[25, 38, 60, 65, 68, 69]	46,367	Linear dose-response^a^	0.84	0.79–0.91	0.0
Apgar Score < 7	5	[39, 56, 60, 65, 69]	805,274	Linear dose-response^a^	0.90	0.69–1.17	65.7
Jaundice	5	[56, 60, 65, 68, 69]	32,109	Linear dose-response^a^	0.71	0.66–0.77	53.7
Respiratory distress syndrome	5	[37, 59, 60, 65, 69]	43,888	Linear dose-response^a^	0.60	0.54–0.67	45.0
Transient tachypnea of the newborn	5	[37, 55, 59, 60, 65]	40,766	Linear dose-response^a^	0.68	0.54–0.86	84.1
Pneumothorax	4	[44, 59, 60, 67]	25,121	Binary (37 + 38 WG vs. ≥39 WG)^c^	0.99	0.03–39.19	72.0
Maternal hysterectomy	2	[37, 52]	18,662	Binary (37 + 38 WG vs. ≥39 WG)^c^	1.10	0.03–39.35	0.0
Maternal blood transfusion	2	[37, 60]	15,162	Binary (37 + 38 WG vs. ≥39 WG)^c^	1.21	0.02–65.67	30.0
**Meta-analyses of subgroups**							
NICU admission only ERCS	4	[37, 39, 54, 60]	792,107	Linear dose-response^a^	0.66	0.65–0.67	0.0
NICU death only repeat CS	2	[37, 40]	497,917	U-Shape^b^	< 39 0.67 ≥39 1.68	0.51–0.87 1.07–2.65	0.0

*NICU* Neonatal Intensive Care Unit; *WG* Week of gestation

^a^ Starting at 37 WG, RR for each additional WG

^b^ Starting at 39 WG, RR for each fewer WG and RR for each additional WG

^c^ Comparison of two timeframes; 37 + 38 WG = 37 + (0–6) + 38 + (0–6), 39 WG = 39 + 0

**Table 4:** GRADE summary of findings

Outcome № of participants (studies)	Relative effect (95% CI)^a^	Anticipated absolute effects (95% CI)			Certainty
				Difference	
NICU admission № of participants: 898,272 (1RCT,13 observational studies)	**RR 0.63** (0.56 to 0.71)	3.3%	**2.1%** (1.9 to 2.3)	**1.2% fewer** (1,5 fewer to 1 fewer)	⨁⨁◯◯ LOW
Neonatal death № of participants: 533,503 (4 observational studies)	n/N IG: 274/25,8080 n/N CG: 160/27,5423 < 39 RR 0.59 (0.43 to 0.83) ≥39 RR 2.09 (1.18 to 3.70)				⨁⨁◯◯ LOW
Respiratory morbidity № of participants: 57,693 (9 observational studies)	**RR 0.64** (0.51 to 0.79)	2.6%	**1.7%** (1.3 to 2.1)	**0.9% fewer** (1.3 fewer to 0.5 fewer)	⨁◯◯◯ VERY LOW
Hospitalization ≥5 days № of participants: 59,331 (5 observational studies)	n/N IG: 2222/24,663 n/N CG: 3289/34,668 < 39 RR 0.52 (0.36 to 0.75) ≥39 RR 2.00 (1.40 to 2.86)				⨁◯◯◯ VERY LOW
Sepsis № of participants: 42,381 (4 observational studies)	n/N IG: 366/20,689 n/N CG: 318/21,692 < 39 RR 0.55 (0.44 to 0.67) ≥39 RR 3.57 (1.87 to 6.78)				⨁⨁◯◯ LOW
Hypoglycemia № of participants: 46,367 (1 RCT, 5 observational studies)	**RR 0.84** (0.79 to 0.91)	1.2%	**1.0%** (1.0 to 1.1)	**0.2% fewer** (0.3 fewer to 0.1 fewer)	⨁⨁⨁◯ MODERATE
Apgar Score < 7 № of participants: 805,274 (5 observational studies)	**RR 0.90** (0.69 to 1.17)	0.5%	**0.5%** (0.4 to 0.6)	**0.1% fewer** (0.2 fewer to 0.1 more)	⨁◯◯◯ VERY LOW
Jaundice № of participants: 32,109 (5 observational studies)	**RR 0.71** (0.66 to 0.77)	2.3%	**1.7%** (1.5 to 1.8)	**0.7% fewer** (0.8 fewer to 0.5 fewer)	⨁⨁◯◯ LOW
RDS № of participants: 43,888 (5 observational studies)	**RR 0.60** (0.54 to 0.67)	0.7%	**0.4%** (0.4 to 0.5)	**0.3% fewer** (03 fewer to 0.2 fewer)	⨁⨁◯◯ LOW
TTN № of participants: 40,766 (5 observational studies)	**RR 0.68** (0.54 to 0.86)	2.5%	**1.7%** (1.4 to 2.2)	**0.8% fewer** (12 fewer to 0.4 fewer)	⨁◯◯◯ VERY LOW
Pneumothorax № of participants: 25,121 (4 observational studies)	**RR 0.99** (0.03 to 39.19)	0.1%	**0.1%** (0.0 to 4.5)	**0.0% fewer** (0.1 fewer to 4.4 more)	⨁◯◯◯ VERY LOW
Maternal death № of participants: 14,865 (1 observational studies)	**RR 0.38** (0.04 to 3.40)	0.0%	**0.0%** (0.0 to 0.2)	**0.0% fewer** (0.0 fewer to 0.1 more)	⨁◯◯◯ VERY LOW
Maternal hysterectomy № of participants: 18,662 (3 observational studies)	**RR 1.10** (0.03 to 39.35)	0.2%	**0.2%** (0.0 to 7.9)	**0.0% fewer** (0.2 fewer to 7.7 more)	⨁◯◯◯ VERY LOW
Maternal blood transfusion№ of participants: 15,162 (2 observational studies)	**RR 1.21** (0.02 to 65.67)	0.8%	**1.0%** (0.0 to 54.0)	**0.2% more** (0.8 fewer to 53.2 more)	⨁◯◯◯ VERY LOW
NICU admission only repeat CS № of participants: 527,941 (4 observational studies)	**RR 0.66** (0.65 to 0.67)	3.0%	**2.0%** (2.0 to 2.0)	**1.0% fewer** (1.1 fewer to 1 fewer)	⨁⨁⨁◯ MODERATE
NICU death only repeat CS № of participants: 497,917 (2 observational studies)	n/N IG: 194/24,1683 n/N CG: 158/25,6234 < 39 RR 0.67 (0.51 to 0.87) ≥39 RR 1.68 (1.07 to 2.65)				⨁◯◯◯ VERY LOW

^a^time response with reference category 39 week of gestation (RR = 1)

*CI* Confidence Interval; *CG* Control group; *IG* Intervention group; *n* Number of events; *N* Number of participants; *RCT* Randomized controlled trial; *RDS* Respiratory distress syndrome; *RR* Relative risk; *TTN* Transient tachypnea of the newborn

### Additional analysis

We performed subgroup analyses for the primary outcomes NICU admission and neonatal death as we observed very high clinical and statistical heterogeneity. We performed a subgroup analysis with the studies that only include women with repeated CS. For the incidence of NICU admission we found a reduction of 34% in the reference group 39 + (0–6) WG by pooling four studies (RR 0.66 95% CI 0.65 to 0.67, I^2^ = 0 moderate quality of evidence). The time-response meta-analysis showed a reduction of neonatal mortality until the 39 + (0–6) WG (RR 0.67 95% CI 0.51 to 0.87, I^2^ = 0 very low quality of evidence) and increasing mortality higher than 39 + 6 WG (RR 1.68 95% CI 1.07 to 2.65, I^2^ = 0 very low quality of evidence). The individual study results can be found in Additional file 1.

The included studies did not supply enough information on first CS to perform subgroup analysis for first CS.

We identified one study examining twin pregnancies with elective CS from 35 + 0 to 41 + 6 WG [66]. Considering the association pattern we decided to compare 35 + 0 to 37 + 6 WG with 38 + 0 to 41 + 6 week. We calculated a RR of 14.01 (95% CI 0.91 to 17.72) for NICU admission (35 + 0 to 37 + 6 WG n/*N* = 13/1378; 38 + 0 to 41 + 6 WG n/*N* = 2/850) and a RR of 0.31 (95% CI 0.03 to 3.40) for neonatal death (35 + 0 to 37 + 6 WG n/*N* = 1/1378; 38 + 0 to 41 + 6 WG n/*N* = 2/850).

The sensitivity analyses using univariate analysis for the primary outcomes NICU admission and neonatal death resulted in an RR of 1.67 (95% CI 1.37 to 2.0, I^2^ = 88%) for NICU admission (see Additional file 1) and an OR of 2.24 (95% CI 0.29 to 17.31, I^2^ = 0) for neonatal death, showing higher risks in early term. For the Funnel plot of NICU admission see Additional file 1.

## Comment

### Main findings

We found that the rate of NICU admission decreases from 37 + 0 WG to 39 + (0–6) WG for elective CS. Risk of bias was serious in all studies and we even identified some with critical risk. The certainty of the evidence according to GRADE is low. The risk for respiratory morbidity in neonates and other postnatal events (jaundice, hypoglycemia) decrease in the same manner. Assuming a U-shaped pattern with 39 + (0–6) WG at the minimum, we found a decreasing risk of death from 37 + 0 to 39 + (0–6) WG and increasing from then on. The certainty of the evidence is low and a sensitivity analysis showed wide confidence intervals diminishing the robustness of results. Similar results were seen in hospitalization of the neonate for more than 5 days and sepsis. Certainty of evidence is very low and low for respiratory morbidity, hospitalization of the neonate for more than 5 days and jaundice and sepsis. Only hypoglycemia showed moderate certainty of the evidence.

Maternal mortality is a very rare event in countries of WHO stratum A [70]. We only found one study considering maternal death. The other maternal outcomes hysterectomy and blood transfusion showed higher event rates in late term but this only seems to be a hint regarding the statistical uncertainty. All studies considering maternal outcomes had serious risk of bias and certainty of evidence was very low.

We found one study examining twin pregnancies. Elective CS was planned more often preterm and in general earlier than singleton pregnancies. We could not pool data with that from singleton pregnancies and cannot draw any conclusion on outcomes from identified data.

For future guidelines and decision making in elective planning of CS there is only sufficient evidence regarding neonatal outcomes. The evidence suggests decreasing NICU admissions in late term, especially in repeated CS. There seems to be a U-shape risk pattern for neonatal death with the minimum at 39 + (0–6) WG. Respiratory morbidity in neonates decreases in late term, still, evidence is uncertain. We cannot draw any conclusion from the findings regarding maternal outcomes.

### Limitations

#### Certainty of evidence

We identified serious risk of bias in all included studies due to the main issues of patient selection, confounding and lack of blinding. None of the cohort studies tried to resolve the issue of allocating pregnancies with less complication to late term groups and pregnancies with more complications to early term groups. Nor did any study report the reasons why women were selected for either group. There are diverse possibilities of confounding, for example ante- and postnatal care may not only differ between institutions but also between women considered for early term CS (increased monitoring) and late term. Also NICU admission policies may vary between institutions. Moreover we assume that the knowledge of early term CS is an indicator supporting NICU admission. As we see in Glavind et al., performing an RCT is possible even if randomization must take place in a short period of about two or 3 weeks (e.g. 38 + (0–6) vs 39 + (0–6) WG) [25].

#### Limitations in the review process

Our review has various limitations. We admit methodological limitations by pooling studies with great heterogeneity. We included any study without differentiating inclusion criteria (e.g. elective CS without any medical indication vs. Elective CS with medical indication), which resulted in high heterogeneity.

We could not use any data from the studies that controlled for confounding because the controlling variables were too heterogeneous. Some studies reported the use of ultrasound for an estimation of the gestational age or a combined method with the date of the last menstruation. Others did not report the method.

We did not differentiate or include this information in our analyses and might have missed on relevant issues. Moreover we pooled outcomes like respiratory morbidity which may differ in their definition of measuring. Furthermore, a broader assessment of maternal adverse events might be more relevant than assessing maternal death due to the rarity of events in the countries we considered in our analysis.

Various outcomes can be considered rather surrogates for neonatal morbidity than of direct importance to the patients, such as NICU admission and hypoglycemia [71]. But nevertheless NICU admission may lead to several negative effects on the development of the neonate and the parental relationship, for example the impact on breastfeeding [72, 73]. As NICU admission is always connected with various stressors it may also negatively affect the long term development of the neonate [74, 75]. Moreover the outcome hypoglycemia is a surrogate for neuronal energy and may affect (longterm) neurological development of the neonate [76, 77].

By constructing meta-analyses for NICU admission we summed up data for all WG ≥37 + 0 to 39 + 6 WG because not all included studies specified later WG and also the linear trend showed no change after 39 + 6 WG. For the other outcomes we ignored missing data in > 39 + 6 WG and let the linear trend continue decrease, remain or even change and further on increase (cubic spline models).

We limited our research to high income countries with very low general and child mortality. Those countries have similar rates of elective CS and comparable reasons for CS (e.g. medical, women’s preference, hospitals preference). We excluded lower WHO strata due to various reasons: General and especially child mortality is higher among other due to worse access to health care, and access to health care also indicates the use of CS, for example in central African regions where health care is limited CS rate is lower than 5%. Meanwhile access to health care and elective CS rate vary within one country in rural areas and areas with more infrastructure reflecting prosperity of the people e.g. China, Middle Eastern countries. As women who receive elective CS in low and middle income countries may vary much more regarding the risk and also backgrounds (education, prosperity, access to healthcare and cultural beliefs), this should be covered in a more precise and separate analysis [20].

## Conclusions

We found that elective CS before the 39 + (0–6) WG lead to more NICU admissions, respiratory morbidity of the neonate and neonatal deaths, though death is rare and increases again after 39 + 6 WG. The decreasing respiratory morbidity is in accordance with the current NICE and RCOG guidelines (refs). Except for repeated CS, evidence is very heterogeneous. Nevertheless one can assume due to the strength of effects performing elective CS in late term is advantageous for neonatal morbidity. Glavind et al. performed a systematic review comparing the 38 + (0–6) and 39 + (0–6) WG for NICU admission, respiratory morbidity and maternal adverse events [78]. They showed similar results in the neonatal outcomes and also did not have enough data on maternal adverse events to make any conclusion. Our results do not differ from the original work for the German ministry of health [16], although our methods differed slightly and we assume a more precise validity of the results owing to the time-response analysis. There is not enough evidence on maternal outcomes to support a decision between early and late term CS. There is a need for more research, especially on maternal outcomes to provide a balanced decision between neonatal and maternal health. Moreover it would be desirable to know more about the reasons that can cause heterogeneity to support patient individual decisions based on pregnancy characteristics, morbidities or maternal characteristics.

### Deviations from the protocol

We deviated from the protocol in the extraction of two outcomes. First we did not extract birth weight of the neonate, as we came to the decision, that early term births have naturally lower birth weight than full term neonates. We neither extracted the outcome maternal adverse events, as they were defined so differently and heterogenic, that we could not see any coherence. e did not request study protocols directly from the authors, as we assumed that the probability that protocols for registry studies were developed is low. As we did not pool maternal mortality we end up not using any beta binomial model for pooling data at all. Furthermore we did not pool adjusted data as adjustment factors were too heterogeneous.

## Supplementary information

**Additional file 1: Appendix A.** Search strategies. **Appendix B.** Included and Excluded Studies. **Appendix C.** Study characteristics of included studies. **Appendix D.** Risk of bias assessment with ROBINS-I. **Appendix E.** Results of individual studies. **Appendix F.** Results of individual studies (graphical illustration). **Appendix G.** Funnel plot for NICU admission. **Appendix H.** Sensitivity Analyses.

## Data Availability

The datasets generated and analyzed during the current study are available from the corresponding author on reasonable request.

## Abbreviations

CI: Confidence interval
CS: Caesarean section
NICU: Neonatal intensive care unit
RCT: Randomized controlled trial
RDS: Respiratory distress syndrome
TTN: Transient tachypnea of the neonate
VBAC: Vaginal birth after caesarean section
WHO: World health organization
WG: Week of gestation
