# Supplementary material for: A systematic review and time-response meta-analysis of the optimal timing of elective caesarean sections for best maternal and neonatal health outcomes
Source: BMC Pregnancy Childbirth. 2020 Jul 8;20:395. doi: 10.1186/s12884-020-03036-1 (PMC7341650; doi:10.1186/s12884-020-03036-1)

Appendix A – Search strategies

Search strategy for MEDLINE:

| (neonatal[tiab] OR neo-natal[tiab] OR maternal[tiab] OR Perinatal[tiab] OR peri-natal[tiab] OR "Perinatal Care"[Mesh] OR “intensive care”[tiab] OR "Intensive Care Units"[Mesh] OR oxygen[tiab] OR bleeding[tiab] OR Apgar[tiab] OR hypoglycemia[tiab] OR "Hypoglycemia"[Mesh] OR .hyperbilirubinemia[tiab] OR "Hyperbilirubinemia, Neonatal"[Mesh] OR “birth weight”[tiab] OR "Birth Weight"[Mesh] OR antibiotic*[tiab] OR respirator*[tiab] OR CPAP[tiab] OR "Continuous Positive Airway Pressure"[Mesh])  AND  (Cesarean[tiab] OR Caesarean[tiab] OR Cesarian[tiab] OR Caesarian[tiab] OR "Cesarean Section"[Mesh] OR CSection[tiab] OR “C Section”[tiab] OR “C Sections”[tiab])  AND  (Timing[tiab] OR late[tiab] OR prior[tiab] OR delayed[tiab] OR time[tiab] OR week*[tiab])  AND  Elective[tiab] |
| --- |

Search strategy Embase

| ('newborn intensive care'/de OR 'intensive care'/de OR 'intensive care unit'/de OR 'perinatal period'/de OR 'depression'/de OR 'oxygen'/de OR 'bleeding'/de OR 'Apgar score'/de OR 'hypoglycemia'/de OR 'hyperbilirubinemia'/de OR 'birth weight'/de OR neonatal:ti,ab OR neo-natal:ti,ab OR maternal:ti,ab OR perinatal:ti,ab OR peri-natal:ti,ab OR "intensive care":ti,ab OR depressi*:ti,ab OR oxygen:ti,ab OR bleeding:ti,ab OR apgar:ti,ab OR hypoglycemi*:ti,ab OR hyperbilirubinemia:ti,ab OR "birth weight":ti,ab OR antibiotic*:ti,ab OR respirator:ti,ab OR Cpap:ti,ab OR "Continuous Positive Airway Pressure":ti,ab)  AND  ('cesarean section'/de OR cesarean:ab,ti OR caesarean:ab,ti OR cesarian:ab,ti OR caesarian:ab,ti OR 'c section':ab,ti OR 'c-sections':ab,ti)  AND  ('time'/de OR timing:ab,ti OR late:ab,ti OR prior:ab,ti OR delayed:ab,ti OR time:ab,ti OR week*:ab,ti)  AND  (elective:ab,ti)  AND  Human/de |
| --- |

Limited to EMBASE and Articles, Articles + press, Reviews

Search strategy Cinahl (via EBSCO)

| MW "Perinatal Care" OR MW "Intensive Care Units, Neonatal" OR MW "Intensive Care, Neonatal" OR MW "Intensive Care Units" OR (TI neonatal OR TI neo-natal OR TI maternal OR TI perinatal OR TI peri-natal OR TI "Intensive Care" OR TI "Intensive Care Units") OR (AB neonatal OR AB neo-natal OR AB maternal OR AB perinatal OR AB peri-natal OR AB "Intensive Care" OR AB "Intensive Care Units") OR MW "Depression Postpartum" OR MW "APGAR Score" OR MW Oxygen OR MW "Postpartum Hemorrhage" OR MW Hypoglycemia OR MW Hyperbilirubinemia OR MW "Hyperbilirubinemia, Neonatal" OR MW "Birth Weight" OR MW Antibiotics OR MW "Continuous Positive Airway Pressure" OR (TI depressi* OR TI oxygen OR TI bleeding OR TI Apgar OR TI hypoglycemi* OR TI hyperbilirubinemia OR TI "Birth Weight" OR TI antibiotic* OR TI respirator* OR TI cpap) OR (AB depressi* OR AB oxygen OR AB bleeding OR AB Apgar OR AB hypoglycemi* OR AB hyperbilirubinemia OR AB "Birth Weight" OR AB antibiotic* OR AB respirator* OR AB cpap)  AND  MW "Cesarean Section" OR MW "Cesarean Section, Elective" OR (TI Cesarean OR TI Caesarean OR TI Cesarian OR TI Caesarian OR TI C-Section OR TI "C Section") OR (AB Cesarean OR AB Caesarean OR AB Cesarian OR AB Caesarian OR AB C-Section OR AB "C Section")  AND  MW Time OR (TI time OR TI timing OR TI late OR TI prior OR TI delayed OR TI week) OR (AB time OR AB timing OR AB late OR AB prior OR AB delayed OR AB week)  AND  Elective |
| --- |

Limited to Academic Journals

Search strategy CENTRAL

| #1 MeSH descriptor: [Perinatal Care] explode all trees  #2 MeSH descriptor: [Intensive Care Units] explode all trees  #3 MeSH descriptor: [Depression, Postpartum] explode all trees  #4 MeSH descriptor: [Hypoglycemia] explode all trees  #5 MeSH descriptor: [Hyperbilirubinemia, Neonatal] explode all trees  #6 MeSH descriptor: [Birth Weight] explode all trees  #7 MeSH descriptor: [Continuous Positive Airway Pressure] explode all trees  #8 (neonatal OR neo-natal OR maternal OR Perinatal OR peri-natal OR “intensive care” OR depressi* OR oxygen OR bleeding OR Apgar OR hypoglycemia OR hyperbilirubinemia OR “birth weight” OR antibiotic* OR respirator* OR CPAP):ti,ab,kw  #9 #1 OR #2 OR #3 OR #4 OR #5 OR #6 OR #7 OR #8  #10 MeSH descriptor: [Cesarean Section] explode all trees  #11 (Cesarean OR Caesarean OR Cesarian OR Caesarian OR C-Section OR “C Section” OR “C Sections”):ti,ab,kw  #12 #10 OR #11  #13 (Timing OR late OR prior OR delayed OR time OR week*):ti,ab,kw  #14 (Elective):ti,ab,kw  #15 #9 AND #12 AND #13 AND #14  #16 (clinicaltrials.gov):so  #17 #15 NOT #16 |
| --- |

Appendix B - Included and Excluded Studies

Table B1 Included studies

| Alderdice, F., et al., Admission to neonatal intensive care with respiratory morbidity following 'term' elective caesarean section. Ir Med J, 2005. 98(6): p. 170-2. |
| --- |
| Bailit JL, Gregory KD, Reddy UM, et al. Maternal and neonatal outcomes by labor onset type and gestational age. Am J Obstet Gynecol 2010; 202:245.e1–245.e12. |
| Balchin I, Whittaker JC, Lamont RF, et al. Timing of planned cesarean delivery by racial group. Obstet Gynecol 2008; 111:659–666 |
| Brookfield, K.F., S.S. Osmundson, and A.B. Caughey, Should delivery timing for repeat cesarean be reconsidered based on dating criteria? J Matern Fetal Neonatal Med, 2017: p. 1-5. |
| Chiossi, G., et al., Timing of delivery and adverse outcomes in term singleton repeat cesarean deliveries. Obstet Gynecol, 2013. 121(3): p. 561-9. |
| Clark SL, Miller DD, Belfort MA, et al. Neonatal and maternal outcomes associated with elective term delivery. Am J Obstet Gynecol 2009; 200:156.e1–156.e4. |
| Doan et al The timing of elective caesarean deliveries and early neonatal outcomes  in singleton infants born 37–41 weeks’ gestation |
| Farchi, S., et al., Timing of repeat elective caesarean delivery and neonatal respiratory outcomes. Arch Dis Child Fetal Neonatal Ed, 2010. 95(1): p. F78. |
| Finn, D., et al., Neonatal outcomes following elective caesarean delivery at term: a hospital-based cohort study. J Matern Fetal Neonatal Med, 2016. 29(6): p. 904-10. |
| Gawlik, S., et al., Timing of elective repeat caesarean does matter: Importance of avoiding early-term delivery especially in diabetic patients. J Obstet Gynaecol, 2015. 35(5): p. 455-60. |
| Glavind, J., et al., Elective caesarean section at 38 weeks versus 39 weeks: neonatal and maternal outcomes in a randomised controlled trial. Bjog, 2013. 120(9): p. 1123-32. |
| Graziosi, G.C.M., et al., Elective caesarean section preferably after at least 38 complete weeks of pregnancy. Nederlands Tijdschrift voor Geneeskunde, 1998. 142(42): p. 2300-2303. |
| Hansen, A.K., et al., Risk of respiratory morbidity in term infants delivered by elective caesarean section: cohort study. Bmj, 2008. 336(7635): p. 85-7. |
| Hoffmire, C.A., et al., Elective delivery before 39 weeks: the risk of infant admission to the neonatal intensive care unit. Matern Child Health J, 2012. 16(5): p. 1053-62. |
| Many, A., et al., Neonatal respiratory morbidity after elective cesarean section. J Matern Fetal Neonatal Med, 2006. 19(2): p. 75-8. |
| Matsuo, K., et al., Is 38 weeks late enough for elective cesarean delivery? International Journal of Gynecology and Obstetrics, 2008. 100(1): p. 90-91. |
| McAlister, B.S., M. Tietze, and S. Northam, Early term birth: the impact of practice patterns on rates and outcomes. West J Nurs Res, 2013. 35(8): p. 1026-42. |
| Melamed N, Hadar E, Keidar L, et al. Timing of planned repeat cesarean delivery after two or more previous cesarean sections: risk for unplanned cesarean delivery and pregnancy outcome. J Matern Fetal Neonatal Med 2014; 27:431–438. |
| Morrison et al Neonatal respiratory morbidity and mode of delivery at term: influence of timing of elective caesarean section. BJOG. 1995. |
| Nakashima, J., et al., Elective Cesarean section at 37 weeks is associated with the higher risk of neonatal complications. Tohoku J Exp Med, 2014. 233(4): p. 243-8. |
| Nir, V., E. Nadir, and M. Feldman, Late better than early elective term Cesarean section. Acta Paediatr, 2012. 101(10): p. 1054-7. |
| Parikh, L., et al., Timing and consequences of early term and late term deliveries. J Matern Fetal Neonatal Med, 2014. 27(11): p. 1158-62. |
| Resende, M.C., L. Santos, and I. Santos Silva, [Neonatal Morbidity in Term Newborns Born by Elective Cesarean Section]. Acta Med Port, 2015. 28(5): p. 601-7. |
| Terada, K., et al., Timing of elective cesarean singleton delivery and neonatal respiratory outcomes at a Japanese perinatal center. J Nippon Med Sch, 2014. 81(4): p. 285-8. |
| Tita, A.T., et al., Timing of elective repeat cesarean delivery at term and neonatal outcomes. N Engl J Med, 2009. 360(2): p. 111-20. |
| Tracy, S.K., M.B. Tracy, and E. Sullivan, Admission of term infants to neonatal intensive care: a population-based study. Birth, 2007. 34(4): p. 301-7. |
| Van den Berg, A., et al., Neonatal respiratory morbidity following elective caesarean section in term infants. A 5-year retrospective study and a review of the literature. Eur J Obstet Gynecol Reprod Biol, 2001. 98(1): p. 9-13. |
| Vidic, Z., et al., Timing of elective cesarean section and neonatal morbidity: a population-based study. J Matern Fetal Neonatal Med, 2016. 29(15): p. 2460-2. |
| Vilchez, G., et al., Maternal race and neonatal outcomes after elective repeat cesarean delivery. J Matern Fetal Neonatal Med, 2014. 27(4): p. 368-71. |
| Vilchez, G., et al., Risk of neonatal mortality according to gestational age after elective repeat cesarean delivery. Arch Gynecol Obstet, 2015. |
| Wilmink, F.A., et al., Neonatal outcome following elective cesarean section beyond 37 weeks of gestation: a 7-year retrospective analysis of a national registry. Am J Obstet Gynecol, 2010. 202(3): p. 250.e1-8. |
| Wilmink, F.A., et al., Neonatal outcome following elective cesarean section of twin pregnancies beyond 35 weeks of gestation. Am J Obstet Gynecol, 2012. 207(6): p. 480.e1-7. |
| Yamazaki, H., et al., Neonatal clinical outcome after elective cesarean section before the onset of labor at the 37th and 38th week of gestation. Pediatr Int, 2003. 45(4): p. 379-82. |
| Zanardo, V., et al., The influence of timing of elective cesarean section on risk of neonatal pneumothorax. J Pediatr, 2007. 150(3): p. 252-5. |
| Zanardo, V., et al., Neonatal respiratory morbidity risk and mode of delivery at term: influence of timing of elective caesarean delivery. Acta Paediatr, 2004. 93(5): p. 643-7. |
| Zanardo, V., et al., The influence of timing of elective cesarean section on neonatal resuscitation risk. Pediatr Crit Care Med, 2004. 5(6): p. 566-70. |

Table 2 Excluded studies

| Abdelazim, I., et al., Impact of antenatal oxytocin infusion on neonatal respiratory morbidity associated with elective cesarean section. Arch Med Sci, 2017. 13(3): p. 629-634. | Population |
| --- | --- |
| Aksoy, H., et al., Blood loss in elective cesarean section: is there a difference related to the type of anesthesia? A randomized prospective study. J Turk Ger Gynecol Assoc, 2015. 16(3): p. 158-63. | Intervention |
| Amorim, M.M., et al., Elective caesarean section at 38 versus 39 weeks of gestation: neonatal and maternal outcomes in a randomised controlled trial Are we trivialising neonatal intensive care unit admissions? Bjog, 2013. 120(13): p. 1702. | Study type |
| Ashton, D.M., Elective delivery at less than 39 weeks. Curr Opin Obstet Gynecol, 2010. 22(6): p. 506-10. | Study type |
| Bailey, B.A., J.G. McCook, and C. Chaires, Burden of elective early-term births in rural Appalachia. South Med J, 2014. 107(10): p. 624-9. | Population |
| Bartolo, S., et al., Why women with previous caesarean and eligible for a trial of labour have an elective repeat caesarean delivery? A national study in France. Bjog, 2016. 123(10): p. 1664-73. | Population |
| Caughey, A.B., Elective induction of labour is associated with decreased perinatal mortality and lower odds of caesarean section at 40 and 41 weeks. Evid Based Med, 2013. 18(3): p. 117-8. | Study type |
| Caughey, A.B., Elective induction of labour is associated with decreased perinatal mortality and lower odds of caesarean section at 40 and 41 weeks. Evid Based Med, 2014. 19(6): p. 236. | Study type |
| Caughey, A.B. Elective caesarean section at 38 weeks versus 39 weeks: Neonatal and maternal outcomes in a randomised controlled trial. Obstetrical and Gynecological Survey, 2014. 69, 3-5 DOI: 10.1097/01.ogx.0000442812.42484.57. | Study type |
| Caughey, A.B., Systematic review and meta-analysis: Elective induction of labour is associated with decreased perinatal mortality and lower odds of caesarean section at 40 and 41 weeks. Evidence-Based Medicine, 2014. 19(6): p. 236. | Study type |
| Cho, Y., et al., Elective ceasarean section at 38 weeks versus 39 weeks: neonatal and maternal outcomes in a randomised controlled trial. Bjog, 2014. 121(13): p. 1748. | Study type |
| Cluver, C., et al. Planned early delivery versus expectant management for hypertensive disorders from 34 weeks gestation to term. Cochrane Database of Systematic Reviews, 2017. DOI: 10.1002/14651858.CD009273.pub2. | Population |
| Dahlen, H.M., et al., Texas medicaid payment reform: Fewer early elective deliveries and increased gestational age and birthweight. Health Affairs, 2017. 36(3): p. 460-467. | Intervention |
| Dietz, H.P., The timing of elective caesarean deliveries and early neonatal outcomes in singleton infants born 37-41 weeks' gestation. Aust N Z J Obstet Gynaecol, 2014. 54(6): p. 602. | Study type |
| Dietz, H.P., The timing of elective caesarean deliveries and early neonatal outcomes in singleton infants born 37-41 weeks' gestation...he timing of elective caesarean deliveries and early neonatal outcomes in singleton infants born 37-41 weeks' gestation. Australian & New Zealand Journal of Obstetrics & Gynaecology, 2014. 54(6): p. 602-602 1p. | Study type |
| Doan, E., K. Gibbons, and D. Tudehope, Re: the timing of elective caesarean deliveries and early neonatal outcomes in singleton infants born 37-41 weeks' gestation. Aust N Z J Obstet Gynaecol, 2014. 54(6): p. 602-3. | Study type |
| Doan, E., K. Gibbons, and D. Tudehope, Re: The timing of elective caesarean deliveries and early neonatal outcomes in singleton infants born 37-41 weeks' gestation...Re: The timing of elective caesarean deliveries and early neonatal outcomes in singleton infants born 37–41 weeks’ gestation. Australian & New Zealand Journal of Obstetrics & Gynaecology, 2014. 54(6): p. 602-603 2p. | Study type |
| Dodd, J.M. and C.A. Crowther, Elective delivery of women with a twin pregnancy from 37 weeks' gestation. Cochrane Database Syst Rev, 2003(1): p. Cd003582. | Duplicate |
| Dodd, J.M., et al., Timing of birth for women with a twin pregnancy at term: a randomised controlled trial. BMC Pregnancy Childbirth, 2010. 10: p. 68. | Population |
| Dodd, J.M., et al., Elective birth at 37 weeks of gestation versus standard care for women with an uncomplicated twin pregnancy at term: The Twins Timing of Birth Randomised Trial. BJOG: An International Journal of Obstetrics and Gynaecology, 2012. 119(8): p. 964-973. | Intervention |
| Dodd, J.M., et al., Elective birth at 37 weeks' gestation for women with an uncomplicated twin pregnancy. Cochrane Database Syst Rev, 2014. 2: p. Cd003582. | Population |
| Dunne, C., et al., Outcomes of Elective Labour Induction and Elective Caesarean Section in Low-risk Pregnancies Between 37 and 41 Weeks' Gestation. Journal of Obstetrics and Gynaecology Canada, 2009. 31(12): p. 1124-1130. | Comparator |
| Easter, S.R., et al., Association of Intended Route of Delivery and Maternal Morbidity in Twin Pregnancy. Obstet Gynecol, 2017. 129(2): p. 305-310. | Comparator |
| Edge, N. and B.W.J. Mol Timing of elective caesarean section: A decision analysis. Journal of paediatrics and child health, 2015. 51, 116 DOI: 10.1111/jpc.12884-7. | Intervention |
| Egić, A., et al., Major risk factors of maternal adverse outcome in women with two or more previous cesarean sections. Vojnosanitetski Pregled, 2016. 73(8): p. 751-756. | Population |
| Ehrenthal, D.B., et al., Neonatal outcomes after implementation of guidelines limiting elective delivery before 39 weeks of gestation. Obstet Gynecol, 2011. 118(5): p. 1047-55. | Population |
| Ertugrul, S., et al., Evaluation of neonatal outcomes in elective repeat cesarean delivery at term according to weeks of gestation. J Obstet Gynaecol Res, 2013. 39(1): p. 105-12. | Population |
| Finn, D., et al., Respiratory adaptation in term infants following elective caesarean section. Arch Dis Child Fetal Neonatal Ed, 2017. | Outcome |
| Finn, D., et al., Respiratory adaptation in term infants following elective caesarean section. Archives of Disease in Childhood: Fetal and Neonatal Edition, 2018. 103(5): p. F417-F427. | Intervention |
| Ganchimeg, T., et al., Optimal Timing of Delivery among Low-Risk Women with Prior Caesarean Section: A Secondary Analysis of the WHO Multicountry Survey on Maternal and Newborn Health. PLoS One, 2016. 11(2): p. e0149091. | Population |
| Glavind, J., Author's reply: elective caesarean section at 38 versus 39 weeks of gestation: neonatal and maternal outcomes in a randomised controlled trial. Bjog, 2013. 120(13): p. 1703-4. | Study type |
| Glavind, J., et al., Authors' reply: Elective ceasarean section at 38 weeks versus 39 weeks: neonatal and maternal outcomes in a randomised controlled trial. Bjog, 2014. 121(13): p. 1748-9. | Study type |
| Glavind, J., et al. Do pregnant women prefer timing of elective cesarean section prior to versus after 39 weeks of gestation? Secondary analyses from a randomized controlled trial. The journal of maternal-fetal & neonatal medicine : the official journal of the European Association of Perinatal Medicine, the Federation of Asia and Oceania Perinatal Societies, the International Society of Perinatal Obstetricians, 2014. 27, 1782-6 DOI: 10.3109/14767058.2013.879707. | Outcome |
| Glavind, J., et al. Timing of elective cesarean section and neonatal morbidity: A randomized controlled trial. International journal of gynaecology and obstetrics, 2012. 119, S358 DOI: 10.1016/S0020-7292%2812%2960707-3. | Study type |
| Glavind, J. and N. Uldbjerg, Elective cesarean delivery at 38 and 39 weeks: neonatal and maternal risks. Curr Opin Obstet Gynecol, 2015. 27(2): p. 121-7. | Study type |
| Glavind, J., et al., Authors' reply: elective caesarean section at 38 versus 39 weeks of gestation: neonatal and maternal outcomes in a randomised controlled trial Are we trivialising neonatal intensive care unit admissions? Bjog, 2013. 120(13): p. 1702-3. | Study type |
| Glavind, J., et al., Authors' reply: Elective caesarean section at 38 versus 39 weeks of gestation: balance between the perceived benefits and potential drawbacks. Bjog, 2014. 121(7): p. 907-8. | Study type |
| Gouyon, J.B., et al., Severe respiratory disorders in term neonates. Paediatr Perinat Epidemiol, 2008. 22(1): p. 22-30. | Population |
| Graziosi, G.C., et al., [Elective cesarean section is preferred after the completion of a minimum of 38 weeks of pregnancy]. Ned Tijdschr Geneeskd, 1998. 142(42): p. 2300-3. | Duplicate |
| Hankins, G.D., S.M. Clark, and M.B. Munn, Cesarean section on request at 39 weeks: impact on shoulder dystocia, fetal trauma, neonatal encephalopathy, and intrauterine fetal demise. Semin Perinatol, 2006. 30(5): p. 276-87. | Study type |
| Hansen, A.K., et al., Elective caesarean section and respiratory morbidity in the term and near-term neonate. Acta Obstet Gynecol Scand, 2007. 86(4): p. 389-94. | Intervention |
| Hourani, M., F. Ziade, and M. Rajab, Timing of planned caesarean section and the morbidities of the newborn. N Am J Med Sci, 2011. 3(10): p. 465-8. | Population |
| Howell, E.A., et al., Association between hospital-level obstetric quality indicators and maternal and neonatal morbidity. Jama, 2014. 312(15): p. 1531-41. | Intervention |
| Hutcheon, J.A., et al., Maternal, care provider, and institutional-level risk factors for early term elective repeat cesarean delivery: a population-based cohort study. Matern Child Health J, 2014. 18(1): p. 22-8. | Outcome |
| Lavery, S. and D. Harvey, Neonatal respiratory morbidity and mode of delivery at term: influence of timing of elective cesarean section. Br J Obstet Gynaecol, 1995. 102(10): p. 843. | Study type |
| Lavoue, V., et al., Caesarean section at term: the relationship between neonatal respiratory morbidity and microviscosity in amniotic fluid. Eur J Obstet Gynecol Reprod Biol, 2013. 169(2): p. 239-43. | Population |
| Lee, S.M., et al., Risk of Emergency Operations, Adverse Maternal and Neonatal Outcomes according to the Planned Gestational Age for Cesarean Delivery. J Korean Med Sci, 2018. 33(7): p. e51. | Population |
| Le Guennec, J.C., et al., Elective delivery and the neonatal respiratory distress syndrome. Can Med Assoc J, 1980. 122(3): p. 307-9. | Intervention |
| Little, S.E., et al., The effect of obstetric practice change to reduce early term delivery on perinatal outcome. J Perinatol, 2014. 34(3): p. 176-80. | Population |
| Lockwood, C.J., Fewer elective early term deliveries, more stillbirths? Contemporary OB/GYN, 2016. 61(5): p. 6-9. | Study type |
| Macallister, K.J., et al., Impact of elective caesarean section on neonatal retrieval in Western Australia during a 12-year period. J Perinatol, 2018. | Intervention |
| Mally, P.V., N.T. Agathis, and S.M. Bailey, Early term infants are at increased risk of requiring neonatal intensive care. World J Pediatr, 2016. 12(1): p. 76-81. | Population |
| Martinez-Nadal, S., et al., [Neonatal morbidity in early-term newborns]. An Pediatr (Barc), 2014. 81(1): p. 39-44. | Population |
| Matsuo, K., K. Shimoya, and T. Kimura, Elective cesarean delivery at 38 weeks' gestation: is the timing too early?...Tita TNA, Landon MB, Spong CY, Lai Y, Leveno KJ, Varner MW et al. Eunice Kennedy Shriver NICHD Maternal fetal medicine units network. Timing of elective repeat cesarean delivery at term and neonatal outcomes. N Engl J Med 2009;360:111-20. Journal of Perinatal Medicine, 2009. 37(5): p. 569-569 1p. | Study type |
| Melhado, L., Even at term, timing of cesarean is linked to adverse outcomes. Perspectives on Sexual and Reproductive Health, 2009. 41(2): p. 130-131. | Study type |
| Middleton, P., et al. Planned early birth versus expectant management (waiting) for prelabour rupture of membranes at term (37 weeks or more). Cochrane Database of Systematic Reviews, 2017. DOI: 10.1002/14651858.CD005302.pub3. | Intervention |
| Mohammed, A.B., A.I. Bayo, and M.F. Abu-Jubara, Timing of elective repeated cesarean delivery in patients with previous two or more cesarean section. J Matern Fetal Neonatal Med, 2013. 26(1): p. 10-2. | Population |
| Mostafa Hefny, S., et al., The neonatal respiratory outcome in relation to timing of elective cesarean section at 38 versus 39week gestation: A single center based study. Egyptian Pediatric Association Gazette, 2013. 61(2): p. 78-82. | Population |
| Muoto, I., et al., Shifting Patterns in Cesarean Delivery Scheduling and Timing in Oregon before and after a Statewide Hard Stop Policy. Health Serv Res, 2018. 53 Suppl 1: p. 2839-2857. | Intervention |
| Nicholl, M.C. and M.A. Cattell, Getting evidence into obstetric practice: appropriate timing of elective caesarean section. Aust Health Rev, 2010. 34(1): p. 90-2. | Intervention |
| Nicoll, A.E., et al., An audit of neonatal respiratory morbidity following elective caesarean section at term. Scott Med J, 2004. 49(1): p. 22-5. | Intervention |
| Ojidu, J.I., Delaying planned caesarean delivery until 39 completed weeks of gestation: the experience of a district general hospital. J Obstet Gynaecol, 1999. 19(2): p. 142-5. | Outcome |
| O'Neill, S.M., et al., Trial of labour after caesarean section and the risk of neonatal and infant death: a nationwide cohort study. BMC Pregnancy Childbirth, 2017. 17(1): p. 74. | Comparator |
| Pallasmaa, N., U. Ekblad, and M. Gissler, Severe maternal morbidity and the mode of delivery. Acta Obstet Gynecol Scand, 2008. 87(6): p. 662-8. | Intervention |
| Phaloprakarn, C., S. Tangjitgamol, and S. Manusirivithaya, Timing of elective cesarean delivery at term and its impact on maternal and neonatal outcomes among Thai and other Southeast Asian pregnant women. J Obstet Gynaecol Res, 2016. | Population |
| Razak, A., et al., Morbidities in Neonates Delivered Electively at Early Term. J Obstet Gynaecol India, 2016. 66(4): p. 248-51. | Population |
| Riskin, A., et al., Does cesarean section before the scheduled date increase the risk of neonatal morbidity? Isr Med Assoc J, 2014. 16(9): p. 559-63. | Comparator |
| Robinson, C.J., et al., Timing of elective repeat cesarean delivery at term and neonatal outcomes: a cost analysis. Am J Obstet Gynecol, 2010. 202(6): p. 632.e1-6. | Duplicate |
| Roy, P. and R. Jose Respiratory morbidity in term infants delivered by elective lower segment caesarean section-a randomized control trial. International Journal of Gynecology and Obstetrics. ( var.pagings), 2015. 131, E289. | Population |
| Ryan, C.A. and P. Hughes, Neonatal respiratory morbidity and mode of delivery at term: influence of timing of elective caesarean section. Br J Obstet Gynaecol, 1995. 102(10): p. 843-4. | Study type |
| Salim, R. and E. Shalev, Health implications resulting from the timing of elective cesarean delivery. Reprod Biol Endocrinol, 2010. 8: p. 68. | Study type |
| Salim, R. and E. Shalev, Timing of elective repeat cesarean delivery at term and maternal perioperative outcomes. Obstet Gynecol, 2011. 117(6): p. 1437; author reply 1437-8. | Study type |
| Salim, R. and E. Shalev, Timing of elective repeat cesarean delivery at term and maternal perioperative outcomes...Obstet Gynecol. 2011 Feb;117(2 Pt 1):280-6. Obstetrics & Gynecology, 2011. 117(6): p. 1437-1437 1p. | Study type |
| Sanu, O., Elective caesarean section at 38 versus 39 weeks of gestation: neonatal and maternal outcomes in a randomised controlled trial. Bjog, 2013. 120(13): p. 1703. | Study type |
| Sholapurkar, S.L., Elective caesarean section at 38 versus 39 weeks of gestation: balance between the perceived benefits and potential drawbacks. Bjog, 2014. 121(7): p. 907. | Study type |
| Stock, S.J., et al., Outcomes of induction of labour in women with previous caesarean delivery: a retrospective cohort study using a population database. PLoS One, 2013. 8(4): p. e60404. | Comparator |
| Tanger, H.L., et al., [Less neonatal morbidity with elective caesarean sections at term: local guideline for elective caesarean section is effective]. Ned Tijdschr Geneeskd, 2010. 154: p. A1201. | Intervention |
| Tanger, H.L., et al., Less neonatal morbidity after an elective cesarean section following a gestation period of 39 weeks. Nederlands Tijdschrift voor Geneeskunde, 2010. 154(26): p. 1224-1228. | Intervention |
| Tita, A.T., What we have learned about scheduling elective repeat cesarean delivery at term. Semin Perinatol, 2016. 40(5): p. 287-90. | Study type |
| Tita, A.T.N., et al., Neonatal outcomes of elective early-term births after demonstrated fetal lung maturity. Am J Obstet Gynecol, 2018. 219(3): p. 296.e1-296.e8. | Intervention |
| Todumrong, N., et al., A comparative study of the spontaneous labor rate in scheduled elective cesarean section at 38 weeks versus 39 weeks of gestation in parturient with previous cesarean section. Journal of the Medical Association of Thailand, 2016. 99: p. S37-S41. | Population |
| Trojano, G., et al., The timing of elective caesarean delivery at term in lombardy: A comparison of 2010 and 2014. Italian Journal of Gynaecology and Obstetrics, 2016. 28(2): p. 48-51. | Outcome |
| Tuuli, M.G. and A.O. Odibo, Neonatal outcomes in relation to timing of repeat cesarean delivery at term. Womens Health (Lond Engl), 2009. 5(3): p. 239-42. | Study type |
| Tzur, T., et al., Timing of elective repeat caesarean section: maternal and neonatal morbidity and mortality. J Matern Fetal Neonatal Med, 2011. 24(1): p. 58-64. | Study type |
| Vidic, Z., et al., Timing of elective cesarean section and neonatal morbidity: a population-based study. Journal of Maternal-Fetal and Neonatal Medicine, 2015. | Duplicate |
| Vilchez, G., et al., Decreased risk of prematurity after elective repeat cesarean delivery in Hispanics. J Matern Fetal Neonatal Med, 2015. 28(2): p. 141-5. | Intervention |
| Wax, J.R., et al., Contribution of elective delivery to severe respiratory distress at term. Am J Perinatol, 2002. 19(2): p. 81-6. | Population |
| Weiniger, C.F., et al., Retrospective cohort study to investigate the impact of timing for term cesarean delivery on maternal and neonatal outcomes. J Matern Fetal Neonatal Med, 2018: p. 1-5. | Intervention |
| Wilmink, F.A., et al., [Timing of elective term caesarean sections; trends in the Netherlands]. Ned Tijdschr Geneeskd, 2014. 158: p. A6951. | Outcome |
| Wilmink, F.A., et al., Timing of elective pre-labour caesarean section: A decision analysis. Aust N Z J Obstet Gynaecol, 2018. | Intervention |
| Yee, W., H. Amin, and S. Wood, Elective cesarean delivery, neonatal intensive care unit admission, and neonatal respiratory distress. Obstet Gynecol, 2008. 111(4): p. 823-8. | Intervention |

Appendix C – Study characteristics of included studies

Table C1 Study characteristics of included studies

| **Study** | **Study design**  **Region**  **Setting**  **Recruitment period** | **Exclusion criteria**  **Patient characteristics** | **Birth mode**  **Time points** | **Outcomes** |
| --- | --- | --- | --- | --- |
| **Alderdice 2005** | Retrospective cohort study  Chart-review of:  The Neonatal Intensive Care Outcomes Research and Evaluation Database  Northern Ireland  Several maternity units in four regional Health Boards  2001-2002 | - **Week 37 (37+0-6)** - N: 208 (neonates N: 237) - **Week 38 (38+0-6)** - N: 897 (neonates N: 928) - **Week 39 (39+0-6)** - N: 1072 (neonates N: 1075) - **Week 40 (40+0-6)** - N: 192 (neonates N: 192) - **Week 41 (41+0-6)** - N: 120 (neonates N: 121) - *Indications for CS all (%)* - Repeat CS: 52 - Maternal request: 24 - Breech presentation: 21 | 37 (37+0-6)  38 (38+0-6)  39 (39+0-6)  40 (40+0-6)  41 (41+0-6) | **Neonatal:**  NICU admission (with respiratory morbidity (TTN, RDS)) |
| **Bailit 2010** | Retrospective cohort study  Chart-review of:  The Consortium on Safe Labor Database  USA  10 institutions  2002-2008 | - Multiple pregnancy - Nonvertex deliveries - Gestational age 34 weeks or 42 weeks - Foetus with anomaly - Pregnancies complicated by placenta praevia or accreta or with a prior uterine scar (caesarean or myomectomy) - Missing onset of labour data - **All weeks:**   N: 3959  *Age y M (SD):* 30.1 (6.6)  *Delivery BMI M (SD):* 34.1 (7.6)  *Ethnicity n (%):*  White: 2019 (51.0)  Black: 841 (21.2)  Hispanic: 660 (16.7)  Asian/Pacific islander: 188 (4.8)  Other: 251(6.3)  *Nulliparous n (%):* 2530 (63.9) | 37  38  39  40  41  42 | **Neonatal:**  NICU admission, asphyxia, ventilation use, sepsis, NICU length of stay  **Maternal:**  Hysterectomy |
| **Balchin 2008** | Prospective cohort study  Collection of data from bookings in 15 maternity units using the St. Mary’s Maternity Information System  England, London  15 maternity units  1988-2000 | - Caesareans before gestational age 36 and after 40 - Unscheduled caesareans - **Week 37 (37+0-6)**   N: 3480  *Ethnicity n (%):*  White: 2834 (81.4)  Asian: 390 (11.2)  Black: 256 (7.4)   - **Week 38 (38+0-6)**   N: 11051  *Ethnicity n (%):*  White: 8935 (80.9)  Asian: 1404 (12.7)  Black: 712 (6.4)   - **Week 39 (39+0-6)**   N: 4580  *Ethnicity n (%):*  White: 3969 (86.7)  Asian: 587 (12.8)  Black: 297 (6.5)   - **Week 40 (40+0-6)**   N: 1780  *Ethnicity n (%):*  White: 1435 (80.6)  Asian: 210 (11.8)  Black: 135 (7.6) | (37+0-6)  (38+0-6)  (40+0-6)  (39+0-6) | **Neonatal:**  Neonatal respiratory morbidity (RDS, TTN) |
| **Brookfield K. F. et al. 2017** | Retrospective cohort study Chart-review of:  National Institute of Child Health and Human Development Maternal foetal Medicine Unit Network registry  USA  19 academic centres  1999-2002 | - CS <37^th^ GW - Multiple pregnancies - First CS - Women with hypertension, preeclampsia, pregestational diabetes - Fetes with congenital anomaly, non-reassuring heart tracing - **Week 37 (37+0-6)**   N: 1593  *Age ≥35 y n (%):* 403 (25.3)  *Ethnicity n (%):*  Non-White: 744 (46.7)   - **Week 38 (38+0-6)**   N: 6662  *Age ≥35 y n (%):* 1587 (23.8)  *Ethnicity n (%):*  Non-White: 3464 (52.0)   - **Week 39 (39+0-6)**   N: 5146  *Age ≥35 y n (%):* 1113 (21.6)  *Ethnicity n (%)*  Non-White: 2187 (42.5)   - **Week 40 (40+0-6)**   N: 1552  *Age ≥35 y n (%):* 286 (18.4)  *Ethnicity n (%):*  Non-White: 530 (34.1)   - **Week 41 (41+0)**   N: 649  *Age ≥35 y n (%):* 101 (15.6)  *Ethnicity n (%):*  Non-White: 161 (24.8) | **Only ERCS**  37 (37+0-6)  38 (38+0-6)  39 (39+0-6)  40 (40+0-6)  >41 (41+0) | **Neonatal:**  *Morbidity* *composite* (NICU admission, hypotonia, meconium aspiration, seizures, need for ventilator support, NEC, RDS, TTN, hypoglycaemia, death)  *Respiratory morbidity* *composite* (RDS, TTN, and/or need for ventilator support in the first 24h) |
| **Chiossi 2013** | Retrospective cohort study Chart-review of:  National Institute of Child Health and Human Development Maternal foetal Medicine Unit Network registry  USA  19 academic centres  1999-2002 | - Multiple pregnancy - Women in early labour undergoing repeated caesarean delivery - Foetus with major congenital anomaly - Women whose ultimate choice of a type of delivery could not be reasonably classified - Infant <500g - **Week 37 (37+0-6)**   N: 1296  *Age y M (SD):* 30.6 (5.6)  *Delivery BMI M (SD)*: 34.1 (7.6)  *Ethnicity n (%):*  Caucasian: 624 (48.1)  Black: 289 (22.3)  Hispanic: 315 (24.3)  Other: 68 (5.3)  *Maternal disease* n (%):* 455 (35.1)  *Prior CS n (%):*  1: 708 (54.6)  2: 419 (32.3)  ≥3: 169 (13.1)  *Married n (%):* 886 (68.4)  *Payer n (%):*  Medicaid or none: 631 (48.7)  Other: 665 (51.3)  *Smoker n (%):* 154 (11.9)   - **Week 38 (38+0-6)**   N: 4601  *Age y M (SD):* 30.6 (5.4)  *Delivery BMI M (SD)*: 33.2 (6.9)  *Ethnicity n (%):*  Caucasian: 2472 (53.7)  Black: 849 (18.5)  Hispanic: 1068 (23.2)  Other: 212 (4.6)  *Maternal disease* n (%)*:  1093(23.8)  *Prior CS n (%):*  1: 2811 (61.1)  2: 1365 (29.7)  ≥3: 425 (9.2)  *Married n (%):* 3339 (72.6)  *Payer n (%):*  Medicaid or none: 1838 (40.0)  Other: 2760 (60.0)  *Smoker n (%):* 512 (11.1)   - **Week 39 (39+0-6)**   N: 6941  *Age y M (SD):* 29.8 (5.4)  *Delivery BMI M (SD)*: 33.6 (6.9)  *Ethnicity n (%):*  Caucasian: 3096 (44.6)  Black: 1475 (21.3)  Hispanic: 2071 (29.8)  Other: 299 (4.3)  *Maternal disease* n (%):* 1383 (19.9)  *Prior CS n (%):*  1: 4200 (60.5)  2: 2207 (31.8)  ≥3: 534 (7.7)  *Married n (%):* 4524 (65.2)  *Payer n (%):*  Medicaid or none: 3525 (50.8)  Other: 3416 (49.2)  *Smoker n (%):* 880 (12.7)   - **Week 40 (40+0-6)**   N: 1492  *Age y M (SD):* 28.7 (5.8)  *Delivery BMI M (SD)*: 33.8 (6.7)  *Ethnicity n (%):*  Caucasian: 491 (32.9)  Black: 387 (25.9)  Hispanic: 540 (36.2)  Other: 74 (5.0)  *Maternal disease* n (%):* 206 (13.8)  *Prior CS n (%)*  1: 1083 (72.6)  2: 332 (22.2)  ≥3: 77 (5.2)  *Married n (%):* 859 (57.6)  *Payer n (%):*  Medicaid or none:936 (62.7)  Other: 556 (37.3)  *Smoker n (%):*182 (12.2)   - **Week 41 (41+0-6)**   N: 535  *Age y M (SD):* 28.1 (5.9)  *Delivery BMI M (SD)*: 34.5 (6.6)  *Ethnicity n (%):*  Caucasian: 127 (23.7)  Black: 138 (25.8)  Hispanic: 237 (44.3)  Other: 33 (6.2)  *Maternal disease* (%):* 54 (10.1)  *Prior CS n (%):*  1: 431 (80.6)  2: 88 (16.4)  ≥3: 16 (3.0)  *Married n (%):* 284 (53.1)  *Payer n (%):*  Medicaid or none:  408 (76.3)  Other: 127 (23.7)  *Smoker n (%):* 54 (10.1) | **Only ERCS**  37 (37+0-6)  38 (38+0-6)  39 (39+0-6)  40 (40+0-6)  41 (41+0-6) | **Neonatal:**  NICU admission, death, RDS, TTN, sepsis, seizure, 5min Apgar score 3 or less  **Maternal:**  Death, blood transfusion, *Composite morbidity* (death, pulmonary oedema, caesarean hysterectomy, pelvic or abdominal abscess, deep vein thrombosis/pulmonary embolism, pneumonia, blood transfusion)  *Maternal disease: Asthma, diabetes, pregestational chronic hypertension with medication, seizure disorder, thyroid disease, renal disease, connective tissue disease |
| **Clark 2009** | Prospective cohort study  Collection of data from bookings in these 27 hospitals  USA  27 hospitals within the Hospital Corporation of America system  05.2007-07.2007 | - Delivery before gestation age 37 - **Week 37(37+0-6)**   N ERCS: 105  N primary CS: 24   - **Week 38 (38+0-6)**   N ERCS: 696  N primary CS: 97   - **Week 39+**   N ERCS: 776  N primary CS: 153 | 37 (37+0-6)  38 (38+0-6)  39+ | **Neonatal:**  NICU admission, birthweight, initial or subsequent admission to a neonate special care unit (defined as any unit other than the normal neonate unit), length of stay in special care unit  **Maternal:**  Initial cervical dilatation, initial blood pressure for women with hypertensive disease as the indication for delivery, agent used for induction, length of labour, route of delivery |
| **Doan 2014** | Retrospective cohort-study Chart-review of:  Mater Mothers’ Hospitals obstetric and neonatal databases  Australia, Queensland  Mater Mothers’ Hospitals  1998-2009 | - Multiple pregnancy - Women with pregnancy conditions (HIV/AIDS, autoimmune disease, hypertension, diabetes) - Neonates with congenital anomalies and stillbirths - **Week 37-38 (37–38+0-6)**   N: 8657  *Age y M (SD):* 32.8 (4.69)  *Delivery BMI* n (%):*  Underweight: 430 (4.9)  Normal: 4557 (52.6)  Overweight: 1790 (20.7)  Obese: 1199 (13.9)  Unknown: 681 (7.9)  *Ethnicity n (%):*  Caucasian: 7905 (91.3)  Indigenous: 57 (0.7)  Asian: 423 (4.9)  Other: 272 (3.1)  *Nulliparous n (%):* 2535 (29.3)  *Primary CS n (%):* 3967 (45.8)  *Indication for CS n (%):*  Foetal: 142 (1.6)  Abnormal presentation: 987 (11.4)  Prior CS: 4655 (53.8)  Prior poor obstetric history: 115 (1.3)  Obstetric complication: 622 (7.2)  Maternal request: 1254 (14.5)  Maternal comorbidity: 594 (6.9)  Unknown: 8 (0.1)  Other: 280 (3.2)  *Marital status n (%):*  Married/living together: 8193 (95.8)  Single: 267 (3.1)  Separated/Divorced/Widowed: 90 (1.1)  *Socioeconomic status n (%):*  Quintile 1: 483 (5.6)  Quintile 2: 223 (2.6)  Quintile 3: 667 (7.7)  Quintile 4: 2483 (28.7)  Quintile 5: 4789 (55.4)  *Private insurance n (%):* 6568 (75.9)  *Smoker n (%):* 624 (7.2)   - **Week 39-41 (39–41+0-6)** - N: 5790   *Age y M (SD):* 32.2 (4.87)  *Delivery BMI* n (%):*  Underweight: 249 (4.3)  Normal: 3097 (53.5)  Overweight: 1184 (20.5)  Obese: 762 (13.2)  Unknown: 498 (8.6)  *Ethnicity n (%):*  Caucasian: 5173 (89.3)  Indigenous: 63 (1.1)  Asian: 319 (5.5)  Other: 235 (4.1)  *Nulliparous n (%):* 1901 (32.8)  *Primary CS n (%):* 2717 (46.9)  *Indication for CS n (%):*  Foetal: 59 (1.0)  Abnormal presentation: 873 (15.1)  Prior CS: 3050 (52.7)  Prior poor obstetric history: 71 (1.2)  Obstetric complication: 346 (6.0)  Maternal request: 854 (14.8)  Maternal comorbidity: 282 (4.9)  Unknown: 2 (0.03)  Other: 253 (4.4)  *Marital status n (%):*  Married/living together: 5384 (94.5)  Single: 269 (4.7)  Separated/Divorced/Widowed: 47 (0.8)  *Socioeconomic status n (%):*  Quintile 1: 346 (6.0)  Quintile 2: 121 (2.1)  Quintile 3: 460 (8.0)  Quintile 4: 1655 (28.6)  Quintile 5: 3201 (55.3)  *Private insurance n (%):* 3727 (64.4)  *Smoker n (%):* 491 (8.5) | 37-38  (37-38+0-6)  39-41  (39-41+0-6) | **Neonatal:**  NICU admission, death, RDS, TTN, pneumothorax, jaundice requiring phototherapy, hypoglycaemia, 5min Apgar score <7, resuscitation (positive pressure ventilation, cardiac compression/ resuscitation drugs), *serious respiratory morbidity*  (NCCU admission with any respiratory diagnosis (RDS, TTN, persistent pulmonary hypertension pneumothorax), birthweight  NICU length of stay ≥1day) |
| **Farchi 2010** | Retrospective cohort-study Chart-review of:  Regional birth and hospital discharge databases.  Italy, Lazio region  Several hospitals  2003-2005 | - Women with chronic and pregnancy-related diseases - Neonates with congenital anomalies and intrauterine foetal growth retardation - **Week 37 (37+0-6)**   N: 2139   - **Week 38 (38+0-6)**   N: 7467   - **Week 39 (39+0-6)**   N: 672   - **Week 40+41(40-41+0-6)**   N: 3051 | 37 (37+0-6)  38 (38+0-6)  39 (39+0-6  40+41 (40-41+0-6) | **Neonatal:**  *Neonatal respiratory morbidity* (pulmonary interstitial emphysema/pneumothorax, RDS, TTN, severe asphyxia (asphyxia and 5-minute Apgar score <7) and other neonatal respiratory problems, and respiratory therapy (need for oxygen and nasal CPAP)) |
| **Finn 2016** | Retrospective cohort-study  Chart-review of:  Theatre logbooks and medical files  Ireland  Cork university maternity hospital  2008-2012 | - Multiple pregnancy - Foetal congenital abnormalities - Delivery took place earlier than planned - Pregnancies warranted early or immediate delivery due to maternal medical or obstetric complications - **Week 37 (37+0-6)**   N: 55  *Age y M (SD):* 33.1 (4.5)  *Parity n (%):*  0: 7 (12.7)  1: 21 (38.2)  2: 14 (25.5)  ≥3: 13 (23.6)  *Indication for CS n (%):*  Prior CS: 37 (67.3)  Prior obstetric complication: 4 (7.3)  Transverse lie: 14 (25.5)  Maternal disease*: 6 (10.9)  Maternal request: 0  Macrosomia (>4.5kg): 0  *Private health insurance n (%):* 23 (41.8)   - **Week 38 (38+0-6)**   N: 576  *Age y M (SD):* 34.5 (4.6)  *Parity n (%):*  0: 71 (12.3)  1: 216 (37.5)  2: 174 (30.2)  ≥3: 115 (20.0)  *Indication for CS n (%):*  Prior CS: 442 (76.7)  Prior obstetric complication: 18 (3.1)  Transverse lie: 68 (11.8)  Maternal disease*: 45 (7.8)  Maternal request: 7 (1.2)  Macrosomia (>4.5kg): 0  *Private insurance n (%):* 307 (53.3)   - **Week 39 (39+0-6)**   N: 2872  *Age y M (SD):* 33.6 (4.5)  *Parity n (%):*  0: 424 (14.8)  1: 1326 (46.2)  2: 813 (28.3)  ≥3: 309 (10.7)  *Indication for CS n (%):*  Prior CS: 2141 (74.6)  Prior obstetric complication: 131 (4.6)  Transverse lie: 495 (17.2)  Maternal disease*: 77 (2.7)  Maternal request: 33 (1.2)  Macrosomia (>4.5kg): 17 (0.6)  *Private insurance n (%):* 1236 (43.0)   - **Week 40 (40+0-6)**   N: 503  *Age y M (SD):* 33.2 (4.6)  *Parity n (%):*  0: 87 (17.3)  1: 283 (56.3)  2: 101 (20.1)  ≥3: 32 (6.3)  *Indication for CS n (%):*  Prior CS: 368 (73.0)  Prior obstetric complication: 15 (3.0)  Transverse lie: 92 (18.3)  Maternal disease*: 11 (2.2)  Maternal request: 5 (1.0)  Macrosomia (>4.5kg): 8 (1.6)  *Private insurance n (%):* 190 (37.8)   - **Week 41 (41+0-6)**   N: 236  *Age y M (SD):* 32.6 (4.6)  *Parity n (%):*  0: 19 (8.1)  1: 189 (80.1)  2: 25 (10.6)  ≥3: 3 (1.2)  *Indication for CS n (%):*  Prior CS: 204 (86.4)  Prior obstetric complication: 3 (1.3)  Transverse lie: 22 (9.31)  Maternal disease*: 1 (0.4)  Maternal request: 0  Macrosomia (>4.5kg): 5 (2.1)  *Private insurance n (%):* 64 (27.1) | 37 (37+0-6)  38 (38+0-6)  39 (39+0-6)  40 (40+0-6) | **Neonatal:**  NICU admission, NICU length of stay ≥2d, RDS, TTN, respiratory morbidity (incidence of TTN, RDS, CPAP combined) antibiotics received, morbidity composite (hypoglycaemia, jaundice, sepsis, 1min Apgar score <7), birth weight  *Maternal disease/condition: includes placenta praevia, back/pelvis problems and fibroids |
| **Gawlik 2015** | Retrospective cohort-study Chart-review of:  Birth registry of the Department  of Obstetrics  Germany, Heidelberg  Heidelberg University Hospital  2006-2011 | - Multiple pregnancy - Foetus with major congenital anomaly - Women with other medical or obstetrical conditions that would warrant early or immediate delivery or influence foetal post-partum performance - Pre-gestational diabetes - Women with onset of labour - Primary CS - **Week 37 (37+0-6)**   N: 226  *Age y M (SD):* 32.88 (5.5)   - **Week 38-41 (41+0-6)**   N: 277  *Age y M (SD):* 33.5 (5.1)  Drop-Outs: n: 14 excluded because of missing values | **Only ERCS**  37 (37+0-6)  38-41 (41+0-6) | **Neonatal:**  NICU admission, birth weight, Apgar Score at 1', 5' and 10' minutes |
| **Glavind 2013** | RCT (open-label)  Denmark  Seven hospitals with neonatal department  2009-2011 | - Multiple pregnancy - Age < 18 years - Language difficulties requiring interpreter - Estimated risk of CS being undertaken before 39+5 weeks - Women with: placenta praevia, high blood pressure, elevated level of liver enzymes, type 1 diabetes/gestational diabetes - Any foetal condition warranting delivery before 39+5 weeks - **Week 38 (38+1-38+5)**   N: 636  *Age y M (SD):* 32.1 (4.4)  *BMI ≥ 30 n (%):* 107 (16.8)  *Nulliparous n (%)*: 126 (19.8)  *Prior CS n (%):*  0: 253 (39.8)  ≥1: 383 (60.2)  *Prior VB n (%):*  0: 426 (67.0)  ≥1: 210 (33.0)  *Indication for CS n (%):*  ≥2 Prior CS: 128 (20.1)  Transverse lie: 114 (17.9)  Maternal disease: 54 (8.5)  Priory complicated birth: 78 (12.3)  Maternal request: 262 (41.2)  *Smoker n (%):* 65 (10)   - **Week 39 (38+6-39+5)**   N: 638  *Age y M (SD):* 31.6 (4.6)  *BMI ≥ 30 n (%):* 111 (17.4)  *Smoker n (%):* 83 (13)  *Nulliparous n (%):* 117 (18.3)  *Prior CS n (%):*  0: 255 (40.0)  ≥1: 383 (60.0)  *Prior VB n (%):*  0: 421 (66.0)  ≥1: 217 (34.0)  *Indication for CS n (%):*  ≥2 Prior CS: 102 (16.0)  Transverse lie: 90 (14.1)  Maternal disease: 57 (8.9)  Priory complicated birth: 83 (13.0)  Maternal request: 306 (48.0)  Drop outs: 1/1 in neonates (stillbirth) | 38 (38+1-38+5)  39 (38+6-39+5) | **Neonatal:**  NICU admission (within 48 hours and within 7 days of delivery)  NICU length of stay ≥2d, any respiratory morbidity, serious respiratory morbidity (mechanical ventilation, or three or more initiated days of treatment with oxygen or CPAP during NICU admission), treated hypoglycaemia, 1min Apgar score ≤7, 5min Apgar score ≤7, antibiotics, birth weight  **Maternal:**  Maternal bleeding needing treatment or transfusion  Maternal composite (death, caesarean hysterectomy, deep vein thrombosis/pulmonary embolism, uterine incision lateral tear, injury to bowel or bladder, procedural or anaesthesia complications, rupture or dehiscence, bleeding needing treatment, re-operation, antibiotics) |
| **Graziosi 1998** | Retrospective cohort-study  Netherlands, Utrecht  Academisch Ziekenhuis Utrecht (AZU) and Wilhelmina Kinderziekenhuis (WKH)  1990-1995 | - Complications**:** preeclampsia, foetal distress, bleeding placenta praevia, congenital anomalies and maternal diseases such as diabetes. - The Caesarean section so could have taken place without problems at any desired time in the full-term periods. - **Weeks <39:**   N: 137  *Indication for CS n (%):*  Breech presentation: 31 (22.6)  Prior CS: 24 (17.5)  Obstetric prehistory: 24 (17.5)  Cephalopelvine disproportion: 19 (13.9)  Maternal disease: 8 (5.8)  Placenta praevia: 9 (6.6)  Foetal transverse position: 4 (2.9)  Others: 18 (13.1)   - **Weeks ≥39:**   N: 135  *Indication for CS n (%):*  Breech presentation: 29 (21.5)  Prior CS: 25 (18.5)  Obstetric prehistory: 25 (18.5)  Cephalopelvine disproportion: 22 (16.3)  Maternal disease: 9 (6.7)  Placenta praevia: 5 (3.7)  Foetal transverse position: 2 (1.5)  Others: 18 (13.3) | 37  38  39  40  ≥41 | **Neonatal:**  NICU admission:  Neonatal morbitity (respiratory morbidity, phototherapy) is defined as morbidity that required NICU admission. Therefore we used the sum of these two outcomes as number of NICU admissions  Respiratory morbidity, Phototherapy, days spent in NICU |
| **Hansen 2008** | Prospective cohort-study Collection of data with birth registration forms and medical records  Denmark, Aarhus  Aarhus University hospital  1998-2006 | - Multiple pregnancy - Pregnancies associated with intrauterine growth retardation, diabetes, pre-eclampsia, hypertension - Neonates with congenital malformations - **All weeks**   *Age <35y n (%):* 28645 (83.1)  *BMI n (%):*  BMI <24: 20399 (59.2)  BMI ≥25: 7421 (21.5)  Missing: 6638 (18.3)  *Delivered by CS n (%):* 2687 (7.8)  Missing: 7044 (20.44)  *Parity n (%):*  0: 16769 (48.7)  ≥1: 17681 (51.3)  *Marital status n (%):*  Married/living together: 25728 (74.7)  Single: 965 (2.8)  Missing: 7765 (22.5)  *No of years of schooling n (%):*  ≥10: 24563 (71.3)  <10: 1980 (5.8)  Missing: 7915 (23.0)  *Smoker (>0 cigarettes/day) n (%):* 3530 (10.2)  Missing: 6541 (19.0)  *Alcohol intake in pregnancy (>1 units/week) n(%):* 23421(11.6) | 37  38  39  40  41 | **Neonatal:**  Respiratory morbidity (any respiratory distress, TTN, persistent pulmonary hypertension), serious respiratory morbidity (≥3 days continuous oxygen supplementation, nasal CPAP, any period of mechanical ventilation) |
| **Many 2006** | Prospective cohort-study  Israel, Tel Aviv | - Multiple pregnancy - Women delivered   before or after booked date   - No documentation of first   semester ultrasound   - **All weeks**   *Age y M (SD):* 32.1 (5.0) | 38  39  40  41 | **Neonatal:**  Neonatal respiratory morbidity (RDS, TTN) |
| **Matsuo 2008** | Prospective cohort-study  Collection of records at Osaka University Hospital  Japan, Osaka  Osaka University Hospital  1994-2005 | - **All weeks**   *Indication for CS (%):*  Prior CS: 51.1  Breech presentation: 23.9  Placenta Previa: 11.3  Drop outs: 15 / 33 / 7 (CS done before scheduled date) | 37  38  ≥39 | **Neonatal:**  RDS, TTN, morbidity (hypocalcaemia, transient hyperbilirubinemia, pneumothorax, neonatal infection (antibiotics), transient thrombocytopenia, hypoglycaemia)  **Maternal:**  Severe morbidity (blood loss >2 L, total abdominal hysterectomy, wound dehiscence, severe adhesions, retained gauze, pyelonephritis, deep vein thrombosis, pulmonary oedema, postpartum cardiomyopathy, angina attack) |
| **McAlister 2013** | Retrospective cohort-study  Chart-review of: Birth certificates of the Texas Department of State Health Services  USA southwest  16 hospitals  2008-2009 | - Multiple pregnancy - Neonates born <37 and >41 weeks - Neonates with major birth anomalies - **All weeks**   *Age y range:* 12-55  *Ethnicity (%):*  White: 23.6  Black: 18.1  Hispanic: 50  *Marital status*  Married (%): 52.2  *Highest education (%):*  Diploma/High school: 43  Baccalaureate: 13  *Payer (%):*  Private insurance: 42  Medicaid: 35.7  Self-pay: 21.8 | 37-38  39-41 | **Neonatal:**  NICU admission |
| **Melamed 2014** | Retrospective cohort study  Chart-review of:  Theatre logbooks and medical files  Israel, Tel Aviv  Medical centre in  Tel Aviv  2010-2011 | - Placenta praevia - Suspected placenta accrete - Maternal cardiac - Renal or lung disease - Major foetal anomalies - Cases scheduled for CS prior to 37+4 weeks - Primary or secondary CS   **Week 38 (37+4 - 38+3)**  N: 264  *Age y M (SD):* 35.0 (3.7)  *>35 y n (%):* 126 (47.7)  *Maternal disease n (%):* Pregestational diabetes: 7 (2.7)  Chronic hypertension: *3* (1.1)  Preeclampsia: 8 (3)  *Foetal Growth Restriction (<10th centile):* 3 (1.1)  *Gravidity median (interquartile range):* 4 (3-5)  *Parity median (interquartile range):* 2 (2-3)  *Number of prior CS median (interquartile range):* 2 (2-2)  2: 206 (78.0)  3: 51 (19.3)  >3: 7 (2.7)  **Week 39 (≥ 38+4)**  N: 113  *Age y M (SD):* 35.1 (3.3)  *>35 years n (%):* 52 (46.0)  *Maternal disease n (%):*  Pregestational diabetes*:* 2 (1.8)  Chronic hypertension*:* 1 (0.9)  Preeclampsia*:* 0 (0.0)  *Foetal Growth Restriction (<10th centile):* 2 (1.8)  *Gravidy median (interquartile range):* 4 (3-5)  *Parity median (interquartile range):* 2 (2-3)  *Number of prior CS median (interquartile range):* 2 (2-2)  2: 90 (79.6)  3: 17 (15.0)  >3: 6 (5.3) | **Only ERCS**  38 (37+4 - 38+3)  39 (≥38+4) | **Neonatal:**  NICU admission*,* respiratory morbidity (RDS, TTN, pneumothorax, CPAP or mechanical ventilation) and *Neonatal composite adverse outcome* (perinatal death, neonatal respiratory morbidity, 5-min Apgar score55, umbilical cord arterial pH57.0, admission to NICU, birth trauma, infectious morbidity, hypoglycaemia or jaundice requiring phototherapy)  **Maternal:**  *Infectious morbidity* (presence of postpartum fever (above 38.0 C, not within the first 24 h after birth), endometritis or any surgical site infection), intestinal or bladder laceration, dehiscence or rupture of uterine scar, accidental extension of uterine scar, need for inverted-T incision, caesarean hysterectomy, postpartum relaparotomy, complications of surgical scar, paralytic ileus, admission to intensive care unit, venous thromboembolism or hospitalization ≥5 d) *composite adverse outcome* (postpartum infectious morbidity, general anaesthesia, postpartum haemorrhage (defined subjectively by the surgeon or by a decrease ≥3 g/dL in post-operative haemoglobin levels), |
| **Morrison 1995** | Prospective cohort-study  Collection of data through delivery unit and obstetrics case records  England, Cambridge  Rosie Maternity Hospital  1985-1993 | - Infants with any evidence of infection including pneumonia or meconium aspiration syndrome | 37  38  39  40  ≥41 | **Neonatal:**  NICU admission with respiratory morbidity (RDS, TTN and oxygen supplimentation) |
| **Nakashima**  **2014** | Retrospective cohort-study  Chart-review of:  Medical and delivery records  Japan, Kansai Region  Kansai Medical University  2006-2012 | - Multiple pregnancy - Pregnancies associated with intrauterine growth retardation, (gestational) diabetes, hypertension, placenta praevia, low-lying placenta, placental abruption, other underlying diseases or obstetrical complications - Prolonged labour, nonreassuring foetal status, premature rupture of membranes or labour onset - Foetal disorders - **Week 37 (37+0-6)**   N: 390  *Age y median (interquartile range):* 35 (31-37)  *Age ≥35y n (%):* 197 (50.5)  *Nulliparous n (%):* 98 (25.1)  *Prior CS n (%):* 273 (70.0)  *Breech presentation n (%):* 88 (22.5)  *History of uterine surgery n (%):* 29 (7.5)   - **Week 38 (38+0-6)**   N: 294  *Age y median (interquartile range):* 34 (31-37)  *Age ≥35y n (%):* 124 (42.2)  *Nulliparous n (%):* 67 (22.8)  *Prior CS n(%):* 210 (71.5)  *Breech presentation n (%):* 62 (21.0)  *History of uterine surgery n (%):* 22 (7.5) | 37 (37+0-6)  38 (38+0-6) | **Neonatal:**  NICU admission, respiratory morbidity (tachypnea, retractions, grunting, cyanosis), CPAP, oxygen supplementation ≥24 h, hypoglycaemia, 5min Apgar score, antibiotics, bacterial infection (defined as the need for antibiotics and an associated serum C-reactive protein level of ≥ 0.25 mg/dL blood samples were also collected for culture), hyperbilirubinemia, birth weight |
| **Nir 2012** | Retrospective cohort-study Chart-review of:  Neonatal and maternal hospital charts  Israel Hadera  Hillel-Yaffe (secondary) medical center  2007-2009 | - Multiple pregnancy - Pregnancies associated with maternal illness (e.g. preeclampsia), intrauterine growth retardation, gestational diabetes, placenta previa - Neonates with congenital defect - Active delivery - Neonates born before completion of 37 weeks - **Week 37-38 (38+0-6)**   N: 596   - **Week ≥39**   N: 454 | 37-38 (38+0-6)  ≥39 | **Neonatal:**  NICU admission (including low Apgar score, hypoglycaemia, dehydration, dyspnoea, jaundice, other) |
| **Parikh 2014** | Retrospective cohort study  Patient reported data of: Participants of the Alere Health program  USA  Several clinics,  pregnancy risk assessment and  education program administered by Alere Health, Inc.  2008-2011 | - Multiple pregnancies - **Week 37**   N: 553  *Age y M (SD):* 32.4 (5.3)  *BMI* ≥*30 (%):* 33.6  *Multiparous (%):* 93.5  *Married (%):*82.5  *Smoker (%):*1.8   - **Week 38**   N: 3971  *Age y M (SD):* 31.8 (4.9)  BMI ≥*30 (%):* 29.9  *Multiparous (%):* 94.4  *Married (%):* 86.8  *Smoker (%):* 2.5   - **Week ≥39**   N: 10089  *Age y M (SD):*  32.0 (4.8)  *Multiparous (%):* 92.8  *BMI ≥30 (%):* 28.8  *Married (%):* 88.0  *Smoker (%):* 1.9  Drop outs (reasons): insurance changes precluding the women from program participation or were lost to follow up | 37  38  ≥39 | **Neonatal:**  NICU admission, death, >5 nursery days |
| **Resende 2015** | Retrospective Cohort-Study  Portugal  1 maternity hospital (level 3)  2003-2013 | - Multiple pregnancy - Pregnancies associated with maternal illness (preeclampsia, poorly controlled diabetes mellitus, Rhisoimmunization), intrauterine growth retardation, premature rupture of membranes, - Neonates with congenital malformations - **Week 37-38 (38+0-6)**   N: 1427  *Age y M (range):*  33 (14-46)  *Age ≥35y n (%):* 495 (35)  *Nulliparous n (%):* 551 (39)  *Gestational diabetes n (%):* 83 (6)  *Type of care n (%):*  Private physicians: 739 (52)  Hospital: 541 (38)  Health centre: 26 (2)  Health centre and hospital: 97 (7)   - **Week 39-41 (41+0-6)**   N: 1696  *Age y M (range):* 33 (16-46)  *Age ≥35y n (%):* 593 (35)  *Nulliparous n (%):* 531 (31)  *Gestational diabetes n (%):* 105 (6)  *Type of care n (%):*  Private physicians: 909 (53)  Hospital: 503 (30)  Health center: 38 (2)  Health center and hospital: 213 (13)   - **All weeks:**   *Indications for CS n (%):*  Prior CS: 1714 (55)  Breech position: 888 (28)  Foetal-pelvic incompatibility: 176 (6)  malformation of the uterus/myomectomy: 51 (1.6)  Macrosomia: 37 (1)  Maternal diseases (e.g. HIV+): 257 (8) | 37-38 (38+0-6)  39-41 (41+0-6) | **Neonatal:**  Respiratory morbidity (TTN, RDS, oxygen supplementation, ventilation), hypoglycaemia <2.8 mmol/l, 1min Apgar score <5, sepsis, hyperbilirubinemia, resuscitation, birth weight <2500g, hospitalization ≥5 d |
| **Terada 2014** | Retrospective cohort study  Chart-review of:  Neonatal and maternal hospital charts  Japan, Tokyo  Red Cross Katsushika Maternity Hospital  2006-2013 | - Labour pains; rupture of membranes; perinatal complications, such as pregnancy-induced hypertension, foetal anomalies, and foetal growth restriction (neonatal birth weight less than -1.5 SD of the reference range in Japan); or inaccurate gestational age. - Caesarean sections upon maternal request - *Neonates without respiratory disorders n: 1810*   *Age y M (SD):* 33.8 (4.8)  *Nulliparous n (%):* 471 (26)  *Indication for CS n (%)*  Prior CS: 1181 (65)  Breech presentation: 209 (12)  Placenta praevia/low- lying Placenta: 121 (6.7)  *Gestational age at delivery w M (SD):* 38.0 (0.6)   - *Neonates with respiratory disorders n: 141*   *Age y M (SD):* 34.0 (4.9)  *Nulliparous n (%):* 23  *Indication for CS n (%):*  Prior CS: 99 (70)  Breech presentation: 21 (15)  Placenta praevia/low-lying Placenta: 6 (4.39)  *Gestational age at delivery w M (SD):* 37.7 (0.4) | 37  38  39-40  41 | **Neonatal:**  Neonatal respiratory disorders (RDS, TTN requiring oxygen supplementation or respiratory support, such as intermittent mandatory ventilation and CPAP) |
| **Tita 2009** | Retrospective cohort study Chart-review of:  National Institute of Child Health and Human Development Maternal foetal Medicine Unit Network registry  USA  19 academic centres  1999-2002 | - Multiple gestations - Foetus with a major congenital anomaly - Women with other medical or obstetrical conditions that would warrant early or immediate delivery.   Maternal:   - **Week 37**   N: 834  *Age y M (SD):* 30,4 (5,5)  *Delivery BMI M (SD)*: 32,8 (7,3)  *Ethnicity n (%)*  White: 427(51.2)  Black: 160 (19,2)  Hispanic: 201 (24,1)  Other: 46 (5,5)  *Diet-controlled gestational diabetes mellitus n(%):* 55 (6,6)  *Prior CS n (%):*  1 prior CS: 444 (53,2)  2 prior CS: 272 (32,6)  ≥3 prior CS: 118 (14,2)  *Married n(%):* 574 (68,8)  *Payer n (%):*  Medicaid: 390 (46,8)  Private insurance: 444 (53,2)  *Smoker (%):* 95 (11,4)   - **Week 38**   N: 3909  *Age y M (SD):* 50,5 (5,4)  *Delivery BMI M (SD)*: 32,8 (6,7)  *Ethnicity n (%)*  White: 2194(56,1)  Black: 637(16,3)  Hispanic: 896 (22,9)  Other: 182 (4,7)  *Diet-controlled gestational diabetes mellitus n (%):*  225 (5,76)  *Prior CS n (%):*  1 prior CS: 2403 (61,5)  2 prior CS: 1135(29,0)  ≥ 3 prior CS: 371(9,5)  *Married n(%):* 2930 (75.0)  *Payer n (%):*  Medicaid: 1453 (37,2)  Private insurance: 2454 (62,8)  *Smoker n (%):* 415(10,6)   - **Week 39**   N: 6512  *Age y M (SD):* 29,8 (5,6)  *Delivery BMI M (SD)*: 33,4 (6,7)  *Ethnicity n (%)*  White: 2955 (45,4)  Black: 1292 (19,8)  Hispanic: 1991 (30,6)  Other: 274 (4,2)  *Diet-controlled gestational diabetes mellitus n (%):* 350 (5,38)  *Prior CS n (%):*  1 prior CS: 3940 (60,5)  2 prior CS: 2070 (31,8)  ≥3 prior CS: 502 (7,7)  *Married n(%):* 4290 (65,9)  *Payer n (%):*  Medicaid: 3270 (50,2)  Private insurance: 3241 (49,8)  *Smoker n (%):* 795 (12,2)   - **Week 40**   N: 1385  *Age y M (SD):* 28,7 (5,7)  *Delivery BMI M (SD)*: 33,8 (6,8)  *Ethnicity n (%)*  White: 472(34,1)  Black: 336(24,3)  Hispanic: 504(36,4)  Other: 73 (5,3)  *Diet-controlled gestational diabetes mellitus n (%):* 52 (3,8)  *Prior CS n (%):*  1 prior CS: 1006 (80)  2 prior CS: 312 (22,5)  ≥ 3 prior CS: 67 (4,8)  *Married n(%):* 820 (59,2)  *Payer n (%):*  Medicaid: 844 (60,9)  Private insurance: 541 (39,1)  *Smoker n(%):* 162(11,7)   - **Week 41**   N: 505  *Age y M (SD):* 27,9 (5,7)  *Delivery BMI M (SD)*: 34,5 (6,7)  *Ethnicity n (%):*  White: 117 (23,2)  Black: 128 (25,4)  Hispanic: 231 (45,7)  Other: 29 (5,7)  *Diet-controlled gestational diabetes mellitus n (%):* 7 (1,4)  *Prior CS n (%):*  1 prior CS: 407 (80,6)  2 prior CS: 83 (16,4)  ≥ 3 prior CS: 15 (3)  *Married n (%):* 269 (53,3)  *Payer n (%):*  Medicaid: 388 (76,8)  Private insurance: 117 (23,2)  *Smoker n (%):* 51 (10,1)   - **Week ≥42**   N: 113  *Age y M (SD):* 27,4 (0,9)  *Delivery BMI M (SD)*: 34,6 (7,1)  *Ethnicity n (%)*  White: 20 (17,7)  Black: 31 (27,4)  Hispanic: 52 (46,0)  Other: 10 (8,9)  *Diet-controlled gestational diabetes mellitus n(%):* 3 (2,7)  *Prior CS n (%):*  1 prior CS: 82 (72,6)  2 prior CS: 26 (23,0)  ≥ 3 prior CS: 5 (4,4)  *Married n(%):* 53 (46,9)  *Payer n (%):*  Medicaid: 90 (9,7)  Private insurance n (%): 23 (20,4)   - *Smoker n (%):* 10 (8,9) | **Only ERCS**  37  38  39  40  41  42 | **Neonatal:**  Hypoglycaemia, composite respiratory morbidity (RDS, TTN), resuscitation (cardiopulmonary resuscitation or ventilator support within 24 hours after birth), birthweight ≤2500g, hospitalisation > 5 days |
| **Tita 2018** | Secondary analysis of an observational study  USA  25 hospitals  2008-2011 | - any medical or obstetrical indications for early delivery (preeclampsia,   eclampsia, gestational hypertension, or complicated chronic hypertension, oligohydramnios,  prior classical, cesarean delivery or prior myomectomy, placenta previa or placenta accreta,  fetal growth restriction, pregestational or gestational diabetes, placental abruption,  chorioamnionitis, premature rupture of membranes, cholestasis of pregnancy,  alloimmunization of pregnancy and fetal or congenital malformations)   - **Week 37-38**   N: 180  *Age y M (range):* 30 (18-56)  *Ethnicity n (%):*  White: 112 (62.2)  Black: 29 (16.1)  Asian: 5 (2.8)  Hispanic: 30 (16.7)  Other: 3 (1.7)  Not specified: 1 (0.6)  *BMI M (range):* *30.6 (21.5-61.8)*  *Parity M (range): 1.0 (0.0-9.0)*  *Smoker n (%):* 23 (12.8)  *Steroids for fetal lung maturity n (%): 6 (3.3)*   - **Week 39-40**   N: 47957  *Age y M (range):*28 (12-57)  *Ethnicity n (%):*  White: 23196 (48.4)  Black: 8762 (18.3)  Asian: 2582 (5.4)  Hispanic: 10851 (22.6)  Other: 2077 (4.3)  Not specified: 489 (1.0)  *BMI M (range):* 29.7 (12.4-99.1)  *Parity M (range):* *1.0 (0.0-11.0)*  *Smoker n (%): 4035 (8.4)*  *Steroids for fetal lung maturity n (%): 228 (0.5)* | 37–38  39–40 | Perinatal death, Ventilation within 24 hours for  ≥2 days, CPAP use, Proven newborn sepsis, Pneumonia, Meningitis, Treated hypoglycaemia, Phototherapy for  Hyperbilirubinemia, Apgar <7 at 5 minutes, Respiratory distress syndrome, Transient tachypnea of the  Newborn, CPR within first 24 hours, Bronchopulmonary dysplasia, Persistent pulmonary  hypertension of the newborn, Necrotizing enterocolitis, IVH, Seizures, NICU admission, NICU stay, NICU stay >2 days, NICU length of stay > 2 (if admitted to nicu), Meconium aspiration, Hypoxic-Ischemic Encephalopathy |
| **Tracy 2007** | Retrospective cohort study  Chart-review of:  National Perinatal Data Collection (data on all births collected from each state and territory and validated by the Australian Institute of Health and Welfare)  Australia  Several hospitals  1999-2002 | - Younger than 20 or older than 34 years - Multiple pregnancies - No birth to a live singleton baby of normal size (10th-90th birthweight percentile) - No presenting in the cephalic position - Not born at term (37-41 week gestation) - Hypertension or diabetes and pregnancy-induced hypertension or gestational diabetes - **Week 37**   N: 7503   - **Week 38**   N: 19984   - **Week 39**   N: 1377   - **Week 40**   N: 5082   - **Week 41**   N: 1733 | 37  38  39  40  41 | **Neonatal:**  NICU admission (refers to admission to any level of neonatal support from special care nursery to NICU, where incubators, oxygen therapy, cardiorespiratory monitoring, intravenous fluid therapy, and tube feeding may be routinely offered. The type of NICU and complex support varies by category or level of unit in Australia) |
| **Van den Berg** | Retrospective  cohort study  Chart-review of:  Neonatal and maternal hospital charts  Netherlands, Amsterdam  University Hospital Vrije Universiteit  1994-1998 | - Complicating factors influencing the timing (ruptured membranes, preceding labour, hypertension, pre-eclampsia, HELLP-Syndrome, signs of maternal infection, foetal distress) - Mothers with diabetes mellitus - Infants with intrauterine growth retardation, congenital malformations - Maternal: n: 324 - Neonatal: n: 333 - **Week 37 (37+0-6)**   N: 95   - **Week 38 (38+0-6)**   N: 183   - **Week ≥39 (39+0)**   N: 55 | 37 (37+0-6)  38 (38+0-6)  ≥39 | **Neonatal:**  NICU admission (for respiratory problems)  Number of hospital days, days in an incubator, ventilation days (oxygen, CPAP, mechanical)  Notes:  Indication for elective caesarean section: breech presentation, social reasons, suspected pelvio-foetal dysproportion, repeat CS, prior fundal scar |
| **Vidic 2016** | National Information Perinatal System (registers all deliveries in Slovenia at 22 GW or when the foetuses weighs 500 g. Registration is mandatory by law)  Slovenia  Several hospitals  Retrospective cohort study  Chart-review of:  2002-2012 | - Multiple pregnancies - Complicated pregnancies - No elective caesarean section (defined as abdominal delivery performed before onset of labour after 37 weeks gestation) - History of any chronic diseases or gestational complications - No clear indication for cesarean section (prior uterine scar or fetal non-cephalic presentation as indication for cesarean section. Cesarean section upon maternal request is not allowed in Slovenia) - **Week 37**   N: 343   - **Week 38**   N: 1753   - **Week 39**   N: 3140   - **Week 40**   N: 1718   - **≥Week 41**   N: 410   - Week <38   Age y M (SD): 31.5 (4.7)   - Week 40   Age y M (SD): 30.3 (4.4) | 37  38  39  40  ≥41 | **Neonatal:**  NICU admission, death hospitalization >5 days,  5 min Apgar score ≤6, RDS (tachypnea, grunting, flaring of the nostrils, retractions and need for supplemental oxygen), hypoglycaemia (plasma glucose level of less than 1.8 mmol/L in the first 24 h of life and less than 2.5mmol/L thereafter), hyperbilirubinemia (need for phototherapy according to nomograms published by American Academy of Pediatrics^1^ |
| **Vilchez 2014** | Retrospective cohort study Chart-review of:  The National Center for Health Statistics-Center for Disease  Control, Natality Database for the United States of America  USA  Several hospitals  2004-2008 | - Multiple pregnancy - No method of delivery repeat cesarean - Multiple pregnancy - attempted trial of labor, - fetal anomalies, - history of diabetes mellitus, and - hypertensive disorders including eclampsia - **Week 36-38** - N (%): 413007 (52) - *Ethnicity n (%):*   African-American 101086 (12.87)  Caucasian (whites) 654 028 (83.28)   - **Week 36-39**   N (%): 699051 (89.01)   - **Week 40**   N (%): 86289 (10.99) | **Only ERCS**  36  37  38  39  40 | **Neonatal:**  NICU admission, 5 min Apgar Score ≤6, surfactant use, antibiotics use, seizures |
| **Vilchez 2015** | Retrospective cohort study  Chart-review of:  The National Center for Health Statistics-Center for Disease  Control, Natality Database for the United States of America  USA  Several hospitals  2004-2008 | - Multiple pregnancies - No method of delivery   by repeat caesarean   - Attempted trial of labour   gestational age at delivery under 37 or over 41   - Any reported congenital anomalies and risk factors such as diabetes, chronic hypertension and pregnancy-related hypertensive disorders including eclampsia. - *Maternal:*   *Ethnicity n (%):*  Whites 394 885 (81,8)  Blacks 59480 (12,3)  American-Indians 2963 (0,61)  Asians 25724 (31)   - *Neonatal:*   *Male n (%): 49*   - **Week 37**   N: 102407   - **Week 38**   N: 264166   - **Week 39**   N: 286044   - **Week 40**   N: 86289   - **Week 41**   N: 36382 | **Only ERCS**  37  38  39  40  41 | **Neonatal:**  Early death (when the demise occurs at less than 7 days), neonatal death (less than 28 days) and infant death (less than 1 year) |
| **Wilmink 2010** | Retrospective cohort study  Chart-review of:  Netherlands Perinatal Registry (hospitals that systematically registered neonatal follow-up)  Netherlands  2000-2006 | - No indication for an elective caesarean section (included repeat caesarean section, breech presentation, traumatic first pregnancy, maternal request) - Pregnancies complicated by intrauterine foetal deaths - Emergency caesarean sections - Multiple pregnancies - Foetus with congenital anomalies - Elective caesarean sections after spontaneous rupture of membranes or signs of labour - Mothers with an adverse medical or obstetric history and/or complications of pregnancy that could influence the risk for neonatal morbidity - **Week 37**   N: 1734  *Age y M (SD):* 32.1 (4.6)  *Ethnicity n (%):*  Western: 1557 (91.5)  Asian: 28 (1.7)  Other: 116 (6.8)  *Nulliparous n (%):* 594 (34.3)  *Position n (%):*  Vertex: 957 (55.2)  Breech: 688 (39.7)  Other: 88 (5.1)  *Socioeconomic status n (%):*  Very high: 359 (21.0)  High: 362 (21.2)  Normal: 331 (19.4)  Low: 336 (19.7)  Very low: 318 (18.6)   - **Week 38**   N: 10139  *Age y M (SD):* 31.9 (4.4)  *Ethnicity n (%):*  Western: 9103 (91.7)  Asian: 146 (1.5)  Other: 677 (6.8)  *Nulliparous n (%):* 2745 (36.6)  *Position n (%):*  Vertex: 4746 (46.9)  Breech: 5029 (49.6)  Other: 355 (3.5)  *Socioeconomic status n (%):*  Very high: 2009 (20.2)  High: 2156 (21.7)  Normal: 1901 (19.1)  Low: 1856 (18.6)  Very low: 2034 (20.4)   - **Week 39**   N: 6647  *Age y M (SD):* 32.0 (4.5)  *Ethnicity n (%):*  Western: 5878 (91.2)  Asian: 109 (1.7)  Other: 459 (7.1)  *Nulliparous n (%):* 2745 (41.3)  *Position n (%):*  Vertex: 2943 (44.3)  Breech: 3434 (51.7)  Other: 266 (4.0)  *Socioeconomic status n (%):*  Very high: 1442 (22.2)  High: 1336 (20.4)  Normal: 1190 (18.2)  Low: 1170 (17.9)  Very low: 1414 (21.6)   - **Week 40**   *N:* 1274  *Age y M (SD):* 31.9 (4.7)  *Ethnicity n (%):*  Western: 1069 (87.2)  Asian: 33 (2.7)  Other: 124 (8.3)  *Nulliparous n (%):* 419 (32.9)  *Position n (%)*  Vertex: 754 (59.3)  Breech: 458 (36.0)  Other: 59 (4.6)  *Socioeconomic status n (%):*  Very high: 237 (18.8)  High: 268 (21.3)  Normal: 224 (17.8)  Low: 221 (17.6)  Very low: 308 (24.5)   - **Week 41**   *N*: 782  *Age y M (SD):* 31.7 (4.5)  *Ethnicity n (%):*  Western: 688 (89.4)  Asian: 10 (1.3)  Other: 72 (9.4)  *Nulliparous n (%):* 295 (37.7)  *Position n (%):*  Vertex: 475 (60.9)  Breech: 279 (35.8)  Other: 26 (3.3)  *Socioeconomic status n (%):*  *V*ery high: 153 (19.8)  High: 184 (23.8)  Normal: 135 (17.5)  Low: 128 (16.6)  Very low: 173 (22.4)   - **≥ Week 42**   N: 397  *Age y M (SD):* 31.7 (4.5)  *Ethnicity n (%):*  Western: 342 (88.4)  Asian: 3 (0.8)  Other: 42 (10.9)  *Nulliparous n (%):* 156 (39.3)  *Position n (%)*  Vertex: 285 (71.8)  Breech: 99 (24.9)  Other: 13 (3.3)  *Socioeconomic status n (%):*  Very high: 71 (17.9)  High: 92 (23.2)  Normal: 81 (20.5)  Low: 65 (16.4)  Very low: 87 (22.0)  Drop outs (reasons): 17852 cases were excluded because of incomplete follow-up | 37  38  39  40  41  ≥42 | **Neonatal:**  NICU admission, death, length of stay ≥5 days, 5-minute Apgar score ≤3, 5-minute Apgar score ≤7, hyperbilirubinemia, necrotizing  enterocolitis, meconium aspiration, composite respiratory outcome and as individual outcomes (RDS, TTN, pneumothorax, air leakage, oxygen, intermittent positive pressure ventilation, CPAP)  Composite outcomes and as individual outcomes (death, severe resuscitation (endotracheal artificial respiration and/or administration of buffers and/or other), sepsis (clinically suspected patients or proven infections with positive cultures), respiratory complications (RDS, wet lung syndrome, TTN pneumothorax, air leakage), respiratory support (oxygen, intermittent positive pressure ventilation, CPAP), hypoglycaemia (defined as a serum or plasma glucose level of 2.5 mmol/L), neurologic morbidity (convulsions or intracranial haemorrhage |
| **Wilmink 2012** | Retrospective cohort study  Chart-review of:  Netherlands Perinatal Registry (hospitals that systematically registered neonatal follow-up)  Netherlands  Several hospitals  2000-2007 | - Singleton pregnancies - No twin pregnancy beyond >35 weeks of gestation - No neonates born by an elective CS >35 weeks of gestation - Neonates born by a planned CS registered with a maternal and/or foetal indication or born by an emergency CS - All twins of which 1 foetus was missing in the registration were excluded - Eventually twins of which at least 1 foetus had a congenital anomaly - Twins of mothers with an adverse medical or obstetric history and/or a complication of pregnancy that could influence the risk for neonatal morbidity - **Week 35**   N: 52 (neonates N: 104)  *Age y M (SD):* 31.4 (5.0)  0-35 y n (%): 38 (73.1)  >35 y n (%): 14 (26.9)  *Ethnicity n (%):*  Western: 48 (92.3)  Other: 4 (7.7)  *Nulliparous n (%):* 30 (57.7)  *Position n(%):*  Vertex: 51(49.0)  Breech: 48(46.2)  Other: 5(4.8)  Missing: 0  *Socioeconomic status n (%):*  Very High: 10 (19.2)  High: 11 (21.2)  Normal: 7 (13.5)  Low: 13 (25.0)  Very low: 11 (21.1)  Missing: 0  *Neonatal* b*irthweight g M (SD):* 2304 (334)   - **Week 36**   N: 154 (Neonate N: 290)  *Age y M (SD):* 32.2 (4.1)  0-35 y n (%): 98 (67.69  >35 y n (%): 47 (32.49  *Ethnicity n (%):*  Western: 132(91.7)  Other: 12(8.3)  Missing (n): 1  *Nulliparous n (%):* 65 (44.8)  Position n (%):  Vertex 104(35.9)  Breech: 164(56.6)  other: 22(7.6)  *Socioeconomic status n (%):*  Very High: 23 (16.3)  High: 33 (23.4)  Normal: 28 (19.9)  Low: 26 (18.4)  Very low: 31 (22.0)  Missing: 4  *Neonatal* b*irthweight g M (SD):* 2521 (379)   - **Week 37**   N: 492 (Neonates N: 984)  *Age y M (SD):* 32.0 (4.4)  0-35 y n (%): 347 (70.5)  >35 y n (%): 145 (29.5)  *Ethnicity n (%):*  Western: 429 (87.6)  Other: 61 (12.4)  Missing: 2  *Nulliparous n (%):* 228 (46.3)  *Position n (%):*  Vertex: 300(30.6)  Breech: 570(58.2)  other: 110(11.2)  missing (n): 4  *Socioeconomic status n (%):*  Very High: 91 (19.0)  High: 105 (21.9)  Normal: 90 (18.8)  Low: 82 (17.1)  Very low: 112 (23.3)  Missing: 12  *Neonatal* b*irthweight g M (SD):* 2521 (379)   - **Week 38+0-41+6**   N: 425 (Neonate N: 850)  *Age y M (SD):* 32.4 (4.7)  0-35 y n (%): 272 (64.0)  >35 y n (%): 153 (36.0)  *Ethnicity n (%):*  Western: 366 (86.7)  Other: 56 (13.3)  Missing: 3  *Nulliparous n (%):* 201 (47.3)  *Position n (%):*  Vertex 298(35.1)  Breech: 471(55.4)  other: 81(9.5)  *Socioeconomic status n (%):*  Very High: 83(19.8)  High SES: 84(20.0)  Normal: 76(18.1)  Low: 69(16.5)  Very low: 107(25.5)  Missing: 6 | 35  36  37  ≥38 | **Neonatal:**  NICU admission, death,  Admission to any neonatal ward for ≥5 days, severe adverse neonatal outcome composite outcome defined as a composite and as individual outcomes (death, 5-minute Apgar score ≤3, convulsions, intracranial haemorrhage, respiratory morbidity (pneumothorax, RDS support by intermittent positive pressure ventilation, severe resuscitation (defined as endotracheal artificial ventilation and/or administration of buffers), sepsis (clinically suspected patients or proven infections with positive cultures)) composite mild morbidity (TTN, respiratory support with CPAP or oxygen, hypoglycaemia (defined as a serum or plasma glucose level of 2.5 mmol/L)) |
| **Yamazaki 2003** | Retrospective cohort study  Japan, Nagaoka  Nagaoka Red Cross Hospital  1998-2000 | - Multiple pregnancies - Pregnancy with placenta praevia - Pregnancy of women who had a diversity of medical complications - **Week 37+0-3**   N: 48  *Indications for CS n (%):*  Breech presentation: 24 (50.0)  Prior CS: 23 (47.9)  Cephalo-pelvic disproportion: 1 (2.1)   - **Week 37+4-38+6**   N: 48  *Indications for CS n (%):*  Breech presentation: 24 (50.0)  Prior CS: 18 (37.5)  Cephalo-pelvic disproportion: 6 (12.5) | 37+0-3  37+4-38+6 | **Neonatal:**  Breathing difficulty (tachypnea or apnea), TTN, apnea, hypoglycaemia, TTN and hypoglycaemia, apnea and hypoglycaemia, low birth weight (≤2500g) |
| **Zanardo 2004A&B** | Retrospective cohort study  Chart-review of:  Neonatal and maternal hospital charts  Italy, Padua  Maternity Department of Padua University (level III centre)  1998-2000 | - Conditions during pregnancy that might increase risk to the   neonate, including acute and chronic maternal illnesses, disorders of pregnancy, foetal abnormalities, foetal distress or potential foetal asphyxia insult, and foetal growth retardation   - **All weeks:**   *Age y M (SD):* 30.9 (2.3)  *Nulliparous (%):* 42  *Indications for CS %:*  Prior CS: 51  Breech presentation: 27  Twin pregnancy: 8  Suspected cephalo-pelvic disproportion: 5  Nulliparous aged 35 years: 2  Fear of labour: 1  In 6% of CS, the indications for delivery were miscellaneous and included other malpresentations, uncomplicated placenta praevia, retinopathy, and myopathies | 37  38  39-41 | **Neonatal:**  TTN, RDS, resuscitation (including intravenous or intratracheal pharmacotherapy, cardiac compression, positive pressure ventilation, oropharyngeal suction, stimulation, drying/warming. Resuscitation in the delivery room was performed according to the International Guidelines for Neonatal Resuscitation. |
| **Zanardo 2007** | Retrospective cohort study  Chart-review of:  Regional Register Database Certificate of Assistance to the birth  Italy, Padua and Verona  Paediatric Departments of Universities Padua and Verona, Level 3 reference centres and 4 Level 3 hospitals  2002-2003 | - **Week 37 (37+0-6)**   N: 1492   - **Week 38 (38+0-6)**   N: 4006   - **Week 39 (39+0-6)**   N: 2490   - **Week 40+41(40-41+0-6)**   N: 2000 | 37(37+0-6)  38 (38+0-6)  39 (39+0-6)  40-41 (40-41+0-6) | **Neonatal:**  Number with pneumothorax  Respiratory morbidity: iatrogenic RDS requiring mechanical ventilation (e.g.pulmonary air leak, pulmonary hypertension, or both). |
| Apgar=appearance, pulse, grimace, activity, respiration; BMI=body mass index; CPAP=continuous positive airway pressure; CPR=cardiopulmonary resuscitation; CS=caesarean section; ERCS=elective repeat caesarean section; GW=gestational week; h=hours; M=mean; NCCU=neonatal critical care unit; NEC=necrotizing enterocolitis; NICU: neonatal intensive care unit; NPIS=National Information Perinatal System; PPV=positive pressure ventilation; TTN=transient tachypnea of new-born; SD=standard deviation; VB=vaginal birth; RCT=randomized controlled trial; RDS= respiratory distress syndrome; PRN=Netherlands Perinatal Registry; IVH=intraventricular haemorrhage y=years; tri US=trimester ultrasound  *Underweight (<18.5 kg/m2); Normal (18.5–24.9 kg/m2); Overweight (25–29.9 kg/m2); Obese (≥30 kg/m2)  ^1^American Academy of Pediatrics Subcommittee on Hyperbilirubinemia. Management of hyperbilirubinemia in the  newborn infant 35 or more weeks of gestation. Pediatrics 2004;114: 297–314. | | | | |

Appendix D - Risk of bias assessment with ROBINS-I

Table D1 Risk of bias assessment with ROBINS-I

| **1.Bias due to confounding** | | | | | | | | | |
| --- | --- | --- | --- | --- | --- | --- | --- | --- | --- |
|  | **1.1 Is there potential for confounding of the effect of intervention in this study?**  If N/PN to 1.1: the study can be considered to be at low risk of bias due to confounding and no further signalling questions need be considered  If Y/PY to 1.1: determine whether there is a need to assess time-varying confounding: | **1.2. Was the analysis based on splitting participants’ follow up time according to intervention received?**  If N/PN, answer questions relating to baseline confounding (1.4 to 1.6)  If Y/PY, go to question 1.3. | Questions relating to baseline confounding only | | | Questions relating to baseline and time-varying confounding | | | **Risk of bias judgement** |
|  |  |  | **1.4. Did the authors use an appropriate analysis method that controlled for all the important confounding domains?** | **1.5. If Y/PY to 1.4: Were confounding domains that were controlled for measured validly and reliably by the variables available in this study?** | **1.6. Did the authors control for any post-intervention variables that could have been affected by the intervention?** | | **1.7. Did the authors use an appropriate analysis method that controlled for all the important confounding domains and for time-varying confounding?** | **1.8. If Y/PY to 1.7: Were confounding domains that were controlled for measured validly and reliably by the variables available in this study?** |  |
| Alderdice 2005 | Y | NA | N | NA | N | | NA | NA | Serious |
| Bailit 2010 | Y | NA | Y | Y | N | | NA | NA | Moderate |
| Balchin 2008 | Y | NA | PY | Y | N | | NA | NA | Moderate |
| Brookfield 2017 | Y | NA | Y | Y | N | | NA | NA | Moderate |
| Chiossi 2013 | Y | NA | Y | Y | N | | NA | NA | Moderate |
| Clark 2009 | Y | NA | N | NA | N | | NA | NA | Serious |
| Doan 2014 | Y | NA | Y | Y | N | | NA | NA | Moderate |
| Farchi 2010 | Y | NA | Y | Y | N | | NA | NA | Moderate |
| Finn 2016 | Y | NA | PY | Y | N | | NA | NA | Moderate |
| Gawlik 2015 | Y | NA | N | NA | N | | NA | NA | Serious |
| Graziosi 1998 | Y | NA | PN | NA | N | | NA | NA | Serious |
| Hansen 2008 | Y | NA | PY | Y | N | | NA | NA | Moderate |
| Many 2006 | Y | NA | Y | Y | N | | NA | NA | Moderate |
| Matsuo 2008 | Y | NA | N | NA | N | | NA | NA | Serious |
| McAllister 2013 | Y | NA | N | NA | N | | NA | NA | Serious |
| Melamed 2014 | Y | NA | Y | Y | N | | NA | NA | Moderate |
| Morrison 1995 | Y | NA | N | NA | N | | NA | NA | Serious |
| Nakashima 2014 | Y | NA | PY | Y | N | | NA | NA | Moderate |
| Nir 2012 | Y | NA | N | NA | N | | NA | NA | Serious |
| Parikh 2014 | Y | NA | N | NA | N | | NA | NA | Serious |
| Resende 2015 | Y | NA | N | NA | N | | NA | NA | Serious |
| Terada 2014 | Y | NA | N | NA | N | | NA | NA | Serious |
| Tita 2009 | Y | NA | Y | Y | N | | NA | NA | Serious |
| Tracy 2007 | Y | NA | PY | Y | N | | NA | NA | Moderate |
| Van den Berg 2001 | Y | NA | N | NA | N | | NA | NA | Serious |
| Vidic 2015 | Y | NA | N | NA | N | | NA | NA | Serious |
| Vilchez 2014 | Y | NA | Y | Y | N | | NA | NA | Moderate |
| Vilchez 2015 | Y | NA | Y | Y | N | | NA | NA | Moderate |
| Wilmink 2010 | Y | NA | Y | Y | N | | NA | NA | Moderate |
| Wilmink 2012 | Y | NA | Y | Y | N | | NA | NA | Moderate |
| Yamazaki | Y | NA | N | NA | N | | NA | NA | Serious |
| Zanardo 2004 | Y | NA | N | NA | N | | NA | NA | Serious |
| Zanardo 2006 | Y | NA | N | NA | N | | NA | NA | Serious |

| **2.Bias in selection of participants into the study** | | | | | | |
| --- | --- | --- | --- | --- | --- | --- |
|  | **2.1. Was selection of participants into the study (or into the analysis) based on participant characteristics observed after the start of intervention?**  If N/PN to 2.1: go to 2.4 | **2.2. If Y/PY to 2.1: Were the post-intervention variables that influenced selection likely to be associated with intervention?** | **2.3 If Y/PY to 2.2: Were the post-intervention variables that influenced selection likely to be influenced by the outcome or a cause of the outcome?** | **2.4. Do start of follow-up and start of intervention coincide for most participants?** | **2.5. If Y/PY to 2.2 and 2.3, or N/PN to 2.4: Were adjustment techniques used that are likely to correct for the presence of selection biases?** | **Risk of bias judgement** |
| Alderdice 2005 | N | NA | NA | Y | NA | Low |
| Bailit 2010 | N | NA | NA | Y | NA | Low |
| Balchin 2008 | N | NA | NA | Y | NA | Low |
| Brookfield 2017 | N | NA | NA | Y | NA | Low |
| Chiossi 2013 | N | NA | NA | Y | NA | Low |
| Clark 2009 | N | NA | NA | Y | NA | Low |
| Doan 2014 | N | NA | NA | Y | NA | Low |
| Farchi 2010 | N | NA | NA | Y | NA | Low |
| Finn 2016 | N | NA | NA | Y | NA | Low |
| Gawlik 2015 | N | NA | NA | Y | NA | Low |
| Graziosi 1998 | N | NA | NA | Y | NA | Low |
| Hansen 2008 | N | NA | NA | Y | NA | Low |
| Many 2006 | N | NA | NA | Y | NA | Low |
| Matsuo 2008 | N | NA | NA | Y | NA | Low |
| McAllister 2013 | N | NA | NA | Y | NA | Low |
| Melamed 2014 | N | NA | NA | Y | NA | Low |
| Morrison 1995 | N | NA | NA | Y | NA | Low |
| Nakashima 2014 | PY | NA | NA | Y | NA | Moderate |
| Nir 2012 | N | NA | NA | Y | NA | Low |
| Parikh 2014 | N | NA | NA | Y | NA | Low |
| Resende 2015 | N | NA | NA | Y | NA | Low |
| Terada 2014 | N | NA | NA | Y | NA | Low |
| Tita 2009 | N | NA | NA | Y | NA | Serious |
| Tracy 2007 | N | NA | NA | Y | NA | Low |
| Van den Berg 2001 | N | NA | NA | Y | NA | Low |
| Vidic 2015 | N | NA | NA | Y | NA | Low |
| Vilchez 2014 | N | NA | NA | Y | NA | Low |
| Vilchez 2015 | N | NA | NA | Y | NA | Low |
| Wilmink 2010 | N | NA | NA | Y | NA | Low |
| Wilmink 2012 | N | NA | NA | Y | NA | Low |
| Yamazaki | N | NA | NA | Y | NA | Low |
| Zanardo 2004 | N | NA | NA | Y | NA | Low |
| Zanardo 2006 | N | NA | NA | Y | NA | Low |

| **3.Bias in classification of interventions** | | | | | | |
| --- | --- | --- | --- | --- | --- | --- |
|  | **3.1 Were intervention groups clearly defined?** | **3.2 Was the information used to define intervention groups recorded at the start of the intervention?** | **3.3 Could classification of intervention status have been affected by knowledge of the outcome or risk of the outcome?** | **3.4 Did entry into the study begin with start of the exposure?** | **3.5 Were exposure assessment methods robust (including methods used to input data)?** | **Risk of bias judgement** |
| Alderdice 2005 | Y | Y | PN | Y | Y | Low |
| Bailit 2010 | Y | Y | PN | Y | Y | Low |
| Balchin 2008 | Y | Y | P | Y | Y | Low |
| Brookfield 2017 | Y | Y | PN | Y | Y | Low |
| Chiossi 2013 | Y | Y | PN | Y | Y | Low |
| Clark 2009 | Y | Y | PN | Y | Y | Low |
| Doan 2014 | Y | Y | PN | Y | Y | Low |
| Farchi 2010 | Y | Y | PN | Y | Y | Low |
| Finn 2016 | Y | Y | PN | Y | Y | Low |
| Gawlik 2015 | Y | Y | PN | Y | Y | Low |
| Graziosi 1998 | Y | Y | N | Y | Y | Low |
| Hansen 2008 | Y | Y | PN | Y | Y | Low |
| Many 2006 | Y | Y | PN | Y | Y | Low |
| Matsuo 2008 | Y | Y | PN | Y | Y | Low |
| McAllister 2013 | Y | Y | PN | Y | Y | Low |
| Melamed 2014 | Y | Y | PN | Y | Y | Low |
| Morrison 1995 | Y | Y | PN | Y | Y | Low |
| Nakashima 2014 | Y | Y | PN | Y | Y | Low |
| Nir 2012 | Y | Y | PN | Y | Y | Low |
| Parikh 2014 | Y | Y | PN | Y | Y | Low |
| Resende 2015 | Y | Y | PN | Y | Y | Low |
| Terada 2014 | Y | Y | N | Y | Y | Low |
| Tita 2009 | Y | Y | PN | Y | Y | Low |
| Tracy 2007 | Y | Y | PN | Y | Y | Low |
| Van den Berg 2001 | Y | Y | PN | Y | Y | Low |
| Vidic 2015 | Y | Y | PN | Y | Y | Low |
| Vilchez 2014 | Y | Y | PN | Y | Y | Low |
| Vilchez 2015 | Y | Y | PN | Y | Y | Low |
| Wilmink 2010 | Y | Y | PN | Y | Y | Low |
| Wilmink 2012 | Y | Y | PN | Y | Y | Low |
| Yamazaki | Y | Y | N | Y | Y | Low |
| Zanardo 2004 | Y | Y | PN | Y | Y | Low |
| Zanardo 2006 | Y | Y | N | Y | Y | Low |

| **4.Bias due to deviations from intended interventions** | | | | | | | | |
| --- | --- | --- | --- | --- | --- | --- | --- | --- |
|  | If your aim for this study is to assess the effect of assignment to intervention, answer questions 4.1 and 4.2 | | | If your aim for this study is to assess the effect of starting and adhering to intervention, answer questions 4.3 to 4.6 | | | | **Risk of bias judgement** |
|  | **4.1. Were there deviations from the intended intervention beyond what would be expected in usual practice?** | **4.2. If Y/PY to 4.1: Were these deviations from intended intervention unbalanced between groups *and* likely to have affected the outcome?** | **4.3. Were important co-interventions balanced across intervention groups?** | | **4.4. Was the intervention implemented successfully for most participants?** | **4.5. Did study participants adhere to the assigned intervention regimen?** | **4.6. If N/PN to 4.3, 4.4 or 4.5: Was an appropriate analysis used to estimate the effect of starting and adhering to the intervention?** |  |
| Alderdice 2005 | N | NA | Y / PY / PN / N / NI | | Y / PY / PN / N / NI | Y / PY / PN / N / NI | NA | Low |
| Bailit 2010 | N | NA | Y / PY / PN / N / NI | | Y / PY / PN / N / NI | Y / PY / PN / N / NI | NA | Low |
| Balchin 2008 | N | NA | Y / PY / PN / N / NI | | Y / PY / PN / N / NI | Y / PY / PN / N / NI | NA | Low |
| Brookfield 2017 | N | NA | Y / PY / PN / N / NI | | Y / PY / PN / N / NI | Y / PY / PN / N / NI | NA | Low |
| Chiossi 2013 | N | NA | Y / PY / PN / N / NI | | Y / PY / PN / N / NI | Y / PY / PN / N / NI | NA | Low |
| Clark 2009 | N | NA | Y / PY / PN / N / NI | | Y / PY / PN / N / NI | Y / PY / PN / N / NI | NA | Low |
| Doan 2014 | N | NA | Y / PY / PN / N / NI | | Y / PY / PN / N / NI | Y / PY / PN / N / NI | NA | Low |
| Farchi 2010 | N | NA | Y / PY / PN / N / NI | | Y / PY / PN / N / NI | Y / PY / PN / N / NI | NA | Low |
| Finn 2016 | N | NA | Y / PY / PN / N / NI | | Y / PY / PN / N / NI | Y / PY / PN / N / NI | NA | Low |
| Gawlik 2015 | N | NA | Y / PY / PN / N / NI | | Y / PY / PN / N / NI | Y / PY / PN / N / NI | NA | Low |
| Graziosi 1998 | N | NA | Y / PY / PN / N / NI | | Y / PY / PN / N / NI | Y / PY / PN / N / NI | NA | Low |
| Hansen 2008 | N | NA | Y / PY / PN / N / NI | | Y / PY / PN / N / NI | Y / PY / PN / N / NI | NA | Low |
| Many 2006 | N | NA | Y / PY / PN / N / NI | | Y / PY / PN / N / NI | Y / PY / PN / N / NI | NA | Low |
| Matsuo 2008 | N | NA | Y / PY / PN / N / NI | | Y / PY / PN / N / NI | Y / PY / PN / N / NI | NA | Low |
| McAllister 2013 | N | NA | Y / PY / PN / N / NI | | Y / PY / PN / N / NI | Y / PY / PN / N / NI | NA | Low |
| Melamed 2014 | N | NA | Y / PY / PN / N / NI | | Y / PY / PN / N / NI | Y / PY / PN / N / NI | NA | Low |
| Morrison 1995 | N | NA | Y / PY / PN / N / NI | | Y / PY / PN / N / NI | Y / PY / PN / N / NI | NA | Low |
| Nakashima 2014 | N | NA | Y / PY / PN / N / NI | | Y / PY / PN / N / NI | Y / PY / PN / N / NI | NA | Low |
| Nir 2012 | N | NA | Y / PY / PN / N / NI | | Y / PY / PN / N / NI | Y / PY / PN / N / NI | NA | Low |
| Parikh 2014 | N | NA | Y / PY / PN / N / NI | | Y / PY / PN / N / NI | Y / PY / PN / N / NI | NA | Low |
| Resende 2015 | N | NA | Y / PY / PN / N / NI | | Y / PY / PN / N / NI | Y / PY / PN / N / NI | NA | Low |
| Terada 2014 | N | NA | Y / PY / PN / N / NI | | Y / PY / PN / N / NI | Y / PY / PN / N / NI | NA | Low |
| Tita 2009 | N | NA | Y / PY / PN / N / NI | | Y / PY / PN / N / NI | Y / PY / PN / N / NI | NA | Low |
| Tracy 2007 | N | NA | Y / PY / PN / N / NI | | Y / PY / PN / N / NI | Y / PY / PN / N / NI | NA | Low |
| Van den Berg 2001 | N | NA | Y / PY / PN / N / NI | | Y / PY / PN / N / NI | Y / PY / PN / N / NI | NA | Low |
| Vidic 2015 | N | NA | Y / PY / PN / N / NI | | Y / PY / PN / N / NI | Y / PY / PN / N / NI | NA | Low |
| Vilchez 2014 | N | NA | Y / PY / PN / N / NI | | Y / PY / PN / N / NI | Y / PY / PN / N / NI | NA | Low |
| Vilchez 2015 | N | NA | Y / PY / PN / N / NI | | Y / PY / PN / N / NI | Y / PY / PN / N / NI | NA | Low |
| Wilmink 2010 | N | NA | Y / PY / PN / N / NI | | Y / PY / PN / N / NI | Y / PY / PN / N / NI | NA | Low |
| Wilmink 2012 | N | NA | Y / PY / PN / N / NI | | Y / PY / PN / N / NI | Y / PY / PN / N / NI | NA | Low |
| Yamazaki | N | NA | Y / PY / PN / N / NI | | Y / PY / PN / N / NI | Y / PY / PN / N / NI | NA | Low |
| Zanardo 2004 | N | NA | Y / PY / PN / N / NI | | Y / PY / PN / N / NI | Y / PY / PN / N / NI | NA | Low |
| Zanardo 2006 | N | NA | Y / PY / PN / N / NI | | Y / PY / PN / N / NI | Y / PY / PN / N / NI | NA | Low |

| **5.Bias due to missing data** | | | | | | |
| --- | --- | --- | --- | --- | --- | --- |
|  | **5.1 Were outcome data available for all, or nearly all, participants?** | **5.2 Were participants excluded due to missing data on intervention status?** | **5.3 Were participants excluded due to missing data on other variables needed for the analysis?** | **5.4 If PN/N to 5.1, or Y/PY to 5.2 or 5.3: Are the proportion of participants and reasons for missing data similar across interventions?** | **5.5 If PN/N to 5.1, or Y/PY to 5.2 or 5.3: Is there evidence that results were robust to the presence of missing data?** | **Risk of bias judgement** |
| Alderdice 2005 | Y | NI | NI | NA | NA | Low |
| Bailit 2010 | Y | NI | NI | NA | NA | Low |
| Balchin 2008 | Y | NI | NI | NA | NA | Low |
| Brookfield 2017 | Y | NI | NI | NA | NA | Low |
| Chiossi 2013 | Y | NI | NI | NA | NA | NI |
| Clark 2009 | Y | NI | NI | NA | NA | Low |
| Doan 2014 | Y | NI | NI | NA | NA | Low |
| Farchi 2010 | Y | NI | NI | NA | NA | Low |
| Finn 2016 | Y | NI | NI | NA | NA | Low |
| Gawlik 2015 | Y | NI | NI | NA | NA | Low |
| Graziosi 1998 | Y | NI | NI | NA | NA | Low |
| Hansen 2008 | Y | NI | NI | NA | NA | Low |
| Many 2006 | Y | NI | NI | NA | NA | Low |
| Matsuo 2008 | Y | NI | NI | NA | NA | Low |
| McAllister 2013 | Y | NI | NI | NA | NA | Low |
| Melamed 2014 | Y | NI | NI | NA | NA | Low |
| Morrison 1995 | Y | NI | NI | NA | NA | Low |
| Nakashima 2014 | Y | NI | NI | NA | NA | Low |
| Nir 2012 | Y | NI | NI | NA | NA | Low |
| Parikh 2014 | Y | NI | NI | NA | NA | Low |
| Resende 2015 | Y | NI | NI | NA | NA | Low |
| Terada 2014 | Y | NI | NI | NA | NA | Low |
| Tita 2009 | Y | NI | NI | NA | NA | Low |
| Tracy 2007 | Y | NI | NI | NA | NA | Low |
| Van den Berg 2001 | Y | NI | NI | NA | NA | Low |
| Vidic 2015 | Y | NI | NI | NA | NA | Low |
| Vilchez 2014 | Y | NI | NI | NA | NA | Low |
| Vilchez 2015 | Y | NI | NI | NA | NA | Low |
| Wilmink 2010 | Y | NI | NI | NA | NA | Low |
| Wilmink 2012 | Y | NI | NI | NA | NA | Low |
| Yamazaki | Y | NI | NI | NA | NA | Low |
| Zanardo 2004 | Y | NI | NI | NA | NA | Low |
| Zanardo 2006 | Y | NI | NI | NA | NA | Low |

| **6. Bias in measurement of outcomes** | | | | | | |
| --- | --- | --- | --- | --- | --- | --- |
|  | **Outcomes assessed** | **6.1 Could the outcome measure have been influenced by knowledge of the intervention received?** | **6.2 Were outcome assessors aware of the intervention received by study participants?** | **6.3 Were the methods of outcome assessment comparable across intervention groups?** | **6.4 Were any systematic errors in measurement of the outcome related to intervention received?** | **Risk of bias judgement** |
| Alderdice 2005 | NICU | Y | Y | Y | PN | Serious |
|  | Respiratory outcomes | PN | Y | PY | PY | Moderate |
| Bailit 2010 | NICU | Y | Y | PY | PN | Serious |
|  | Sepsis | N | Y | PY | PN | Moderate |
|  | (M) Hysterectomy | N | Y | Y | N | Low |
| Balchin 2008 | Respiratory outcomes | PN | Y | PY | PN | Moderate |
| Brookfield 2017 | Respiratory outcomes | PN | Y | PY | PN | Moderate |
| Chiossi 2013 | NICU | Y | Y | Y | PN | Serious |
|  | Apgar score | PY | Y | Y | PN | Serious |
|  | Death | N | Y | Y | N | Low |
|  | Respiratory outcomes, sepsis | PN | Y | Y | PN | Moderate |
|  | (M) Death, hysterectomy | N | Y | Y | N | Low |
|  | (M) Bleeding | Y | Y | Y | PN | Serious |
| Clark 2009 | NICU | Y | Y | Y | PN | Serious |
| Doan 2014 | NICU | Y | Y | Y | PN | Serious |
|  | Apgar Score, jaundice | PY | Y | Y | PN | Serious |
|  | Death, hypoglycemia | N | Y | Y | N | Low |
|  | Respiratory outcomes | PN | Y | PY | PN | Moderate |
| Farchi 2010 | Respiratory outcomes | PN | Y | PY | PN | Moderate |
| Finn 2016 | NICU | Y | Y | Y | PN | Serious |
|  | Respiratory outcomes | PN | Y | PY | PN | Moderate |
| Gawlik 2015 | NICU | Y | Y | Y | PN | Serious |
|  | Apgar score | PY | Y | Y | PN | Serious |
| Graziosi 1998 | NICU | Y | Y | Y | PN | Serious |
|  | jaundice | PY | Y | Y | PN | Serious |
|  | Respiratory outcomes | PN | Y | PY | PN | Moderate |
| Hansen 2008 | Respiratory outcomes | PN | Y | PY Y | PN | Moderate |
| Many 2006 | NICU | Y | Y | Y | PN | Serious |
| Matsuo 2008 | Respiratory outcomes | PN | Y | PY | PN | Moderate |
| McAllister 2013 | NICU | Y | Y | Y | PN | Serious |
| Melamed 2014 | NICU | Y | Y | Y | PN | Serious |
|  | Apgar Score, jaundice | PY | Y | Y | PN | Serious |
|  | Death, hypoglycemia | N | Y | Y | N | Low |
|  | Respiratory outcomes | PN | Y | Y | PN | Moderate |
|  | (M) Hysterectomy | N | Y | Y | PN | Low |
|  | (M) bleeding | Y | Y | Y | PN | Serious |
| Morrison 1995 | Respiratory outcomes | PN | Y | PY | PN | Moderate |
| Nakashima 2014 | NICU | Y | Y | Y | PN | Serious |
|  | jaundice | PY | Y | Y | PN | Serious |
|  | Respiratory outcomes | PN | Y | PY | PN | Moderate |
|  | Hypoglycemia, sepsis | N | Y | PY | PN | Low |
| Nir 2012 | NICU | Y | Y | PY | PN | Serious |
|  | Apgar score, jaundice | PY | Y | Y | PN | Serious |
|  | Respiratory outcomes | PN | Y | PY | PN | Moderate |
|  | Hypoglycemia | N | Y | PY | PN | Low |
| Parikh 2014 | NICU | Y | Y | PY | PN | Serious |
|  | Death | N | Y | Y | N | Low |
| Resende 2015 | NICU | Y | Y | PY | PN | Serious |
|  | Respiratory outcomes | PN | Y | Y | PN | Moderate |
|  | Hypoglycemia | N | Y | Y | PN | Low |
| Terada 2014 | Respiratory outcomes | N | Y | Y | PN | Moderate |
| Tita 2009 | NICU | Y | Y | Y | PN | Serious |
|  | Respiratory outcomes | PN | Y | Y | PN | Moderate |
|  | Hypoglycemia, sepsis | N | Y | Y | PN | Low |
| Tracy 2007 | NICU | Y | Y | Y | PN | Serious |
| Van d. Berg 2001 | NICU | Y | Y | Y | PN | Serious |
|  | Respiratory outcomes | PN | Y | PY | PN | Moderate |
| Vidic 2016 | NICU | Y | Y | Y | PN | Serious |
|  | Apgar score, jaundice | PY | Y | Y | PN | Serious |
|  | Respiratory outcomes | PN | Y | PY | PN | Moderate |
|  | Hypoglycemia | N | Y | Y | PN | Low |
| Vilchez 2014 | NICU | Y | Y | Y | PN | Serious |
|  | Apgar score | PY | Y | Y | PN | Serious |
| Vilchez 2015 | Death | N | Y | Y | N | Low |
| Wilmink 2010 | NICU | Y | Y | Y | PN | Serious |
|  | Apgar score, jaundice | PY | Y | Y | PN | Serious |
|  | Death | N | Y | Y | N | Low |
|  | hypoglycemia, sepsis | N | Y | Y | PN | Low |
|  | Respiratory outcomes | PN | Y | Y | PN | Moderate |
| Wilmink 2012 | NICU | Y | Y | Y | PN | Serious |
|  | Apgar score | PY | Y | Y | PN | Serious |
|  | Death | N | Y | Y | N | Low |
|  | hypoglycemia, sepsis | N | Y | Y | PN | Low |
|  | Respiratory outcomes | PN | Y | PY | PN | Moderate |
| Yamazaki 2003 | Respiratory outcomes | PN | Y | PY | PN | Moderate |
|  | Hypoglycemia | N | Y | Y | PN | Low |
| Zanardo 2004 | Respiratory outcomes | PN | Y | PY | PN | Moderate |
| Zanardo 2007 | Respiratory outcomes | PN | Y | PY | PN | Moderate |

| **7. Bias in selection of the reported result** | | | | |
| --- | --- | --- | --- | --- |
|  | **Is the reported effect estimate likely to be selected, on the basis of the results, from...** | | |  |
|  | **7.1. ... multiple outcome *measurements* within the outcome domain?** | **7.2 ... multiple analyses of the intervention-outcome relationship??** | **57.3 ... different subgroups?** | **Risk of bias judgement** |
| Alderdice 2005 | N | N | N | Low |
| Bailit 2010 | N | N | N | Low |
| Balchin 2008 | N | N | N | Low |
| Brookfield 2017 | N | N | N | Low |
| Chiossi 2013 | N | N | N | NI |
| Clark 2009 | N | N | N | Low |
| Doan 2014 | N | N | N | Low |
| Farchi 2010 | N | N | N | Low |
| Finn 2016 | N | N | N | Low |
| Gawlik 2015 | N | N | N | Low |
| Graziosi 1998 | N | N | N | Low |
| Hansen 2008 | N | N | N | Low |
| Many 2006 | N | N | N | Low |
| Matsuo 2008 | N | N | N | Low |
| McAllister 2013 | N | N | N | Low |
| Melamed 2014 | N | N | N | Low |
| Morrison 1995 | N | N | N | Low |
| Nakashima 2014 | N | N | N | Low |
| Nir 2012 | N | N | N | Low |
| Parikh 2014 | N | N | N | Low |
| Resende 2015 | N | N | N | Low |
| Terada 2014 | N | N | N | Low |
| Tita 2009 | N | N | N | Low |
| Tracy 2007 | N | N | N | Low |
| Van den Berg 2001 | N | N | N | Low |
| Vidic 2015 | N | N | N | Low |
| Vilchez 2014 | N | N | N | Low |
| Vilchez 2015 | N | N | N | Low |
| Wilmink 2010 | N | N | N | Low |
| Wilmink 2012 | N | N | N | Low |
| Yamazaki | N | N | N | Low |
| Zanardo 2004 | N | N | N | Low |
| Zanardo 2006 | N | N | N | Low |

Appendix E - Results of individual studies

Table E1 Results for NICU admission

| **Study** | **37 WG** | | **38 WG** | | **≥39 WG** | |
| --- | --- | --- | --- | --- | --- | --- |
|  | ***Admissions/ Total*** | ***%*** | ***Admissions/ Total*** | ***%*** | ***Admissions/ Total*** | ***%*** |
| Alderdice 2005 | 52/237 | 21.9 | 97/928 | 10.5 | 61/1388 | 4.4 |
| Bailit 2010 | 99/480 | 20.6 | 97/1012 | 9.6 | 174/1928 | 9.0 |
| Chiossi 2013 | 212/1296 | 16.4 | 474/4601 | 10.3 | 620/8968 | 6.9 |
| Clark 2009 | 26/129 | 20.2 | 74/793 | 9.3 | 74/929 | 8.0 |
| Finn 2016 | 12/55 | 21.8 | 79/576 | 13.7 | 332/3611 | 9.2 |
| Glavind 2013 | - | - | 88/635 | 13.9 | 76/637 | 11.9 |
| Graziosi 1998 | 5/44 | 11.4 | 8/95 | 8.4 | 1/133 | 0.8 |
| Melamed 2014 | - | - | 21/264 | 8.0 | 8/113 | 0.7 |
| Parikh 2014 | 63/553 | 11.4 | 182/3971 | 4.6 | 343/10089 | 3.4 |
| Resende 2015 | 14/323 | 4.3 | 28/1104 | 2.5 | 21/1696 | 1.2 |
| Tracy 2007 | 766/7503 | 10.2 | 2120/19984 | 10.6 | 1810/ 20572 | 8.8 |
| Vilchez 2014 | 7125/102407 | 6.7 | 9207/264166 | 3.5 | 12014/408715 | 2.9 |
| Vidic 2016 | 30/ 343 | 8.7 | 41/1753 | 2.3 | 85/5268 | 1.6 |
| Wilmink 2010 | 13/1734 | 0.7 | 32/10139 | 0.3 | 19/9100 | 0.2 |

Table E2 Results for neonatal death

| **Study** | **37 WG** | | **38 WG** | | **39 WG** | | **40 WG** | | **41/42 WG** | |
| --- | --- | --- | --- | --- | --- | --- | --- | --- | --- | --- |
|  | ***Death/Total*** | ***%*** | ***Death /Total*** | ***%*** | ***Death /Total*** | ***%*** | ***Death /Total*** | ***%*** | ***Death /Total*** | ***%*** |
| Chiossi 2013 | 1/1296 | 0.07 | 9/4601 | 0.20 | 4/6941 | 0.06 | 3/1492 | 0.20 | 2/535 | 0.37 |
| Parikh 2014 | 0/553 | 0 | 79/  3971 | 1.99 | 0/  10089 | - | - | - | - | - |
| Vilchez 2015 | 67/  65327 | 0.10 | 117/  170459 | 0.07 | 94/  171316 | 0.05 | 38/  53297 | 0.07 | 17/  22653 | 0.08 |
| Wilmink 2010 | 1/1734 | 0.08 | 0/  10139 | 0 | 1/6647 | 0.02 | 0/1274 | 0 | 1/1179 | 0.08 |

Table E3 Results for maternal death

| **Study** | 37 WG | | 38 WG | | 39 WG | | 40 WG | | 41/42 WG | |
| --- | --- | --- | --- | --- | --- | --- | --- | --- | --- | --- |
|  | ***Death*** **/Total** | % | ***Death*** **/Total** | % | ***Death*** **/Total** | % | ***Death*** **/Total** | % | ***Death*** **/Total** | % |
| Chiossi 2013 | 0/1296 | 0 | 1/4601 | 0.02 | 4/6941 | 0.06 | 0/1492 | 0 | 0/535 | 0 |

Table E4 Results of subgroup analysis for NICU admission repeated CS

| **Study** | **37 WG** | | **38 WG** | | **≥39 WG** | |
| --- | --- | --- | --- | --- | --- | --- |
|  | ***Events/Total*** | ***%*** | ***Admissions/Total*** | ***%*** | ***Admissions/Total*** | ***%*** |
| Chiossi 2013 | 212/1296 | 16.4 | 474/4601 | 10.3 | 620/8968 | 6.9 |
| Clark 2009 | 21/105 | 20.2 | 58/696 | 9.3 | 62/776 | 8.0 |
| Melamed 2014 | - | - | 21/264 | 8.0 | 8/113 | 0.7 |
| Vilchez 2014 | 7125/102407 | 6.7 | 9207/264166 | 3.5 | 12014/408715 | 2.9 |

Table E5 Results of subgroup analysis for neonatal death repeated CS

| **Study** | **37 WG** | | **38 WG** | | **39 WG** | | **40 WG** | | **41/42 WG** | |
| --- | --- | --- | --- | --- | --- | --- | --- | --- | --- | --- |
|  | ***Death/Total*** | ***%*** | ***Death /Total*** | ***%*** | ***Death /Total*** | ***%*** | ***Death /Total*** | ***%*** | ***Death /Total*** | ***%*** |
| Chiossi 2013 | 1/1296 | 0.07 | 9/4601 | 0.20 | 4/6941 | 0.06 | 3/1492 | 0.20 | 2/535 | 0.37 |
| Vilchez 2015 | 67/  65327 | 0.10 | 117/  170459 | 0.07 | 94/  171316 | 0.05 | 38/  53297 | 0.07 | 17/  22653 | 0.08 |

Appendix F - Results of individual studies (graphical illustration)

Figure F1 Neonatal intensive care unit admission

Figure F2 Neonatal death

Appendix G - Funnel plot for NICU admission

Figure G1 Funnel plot for NICU admission


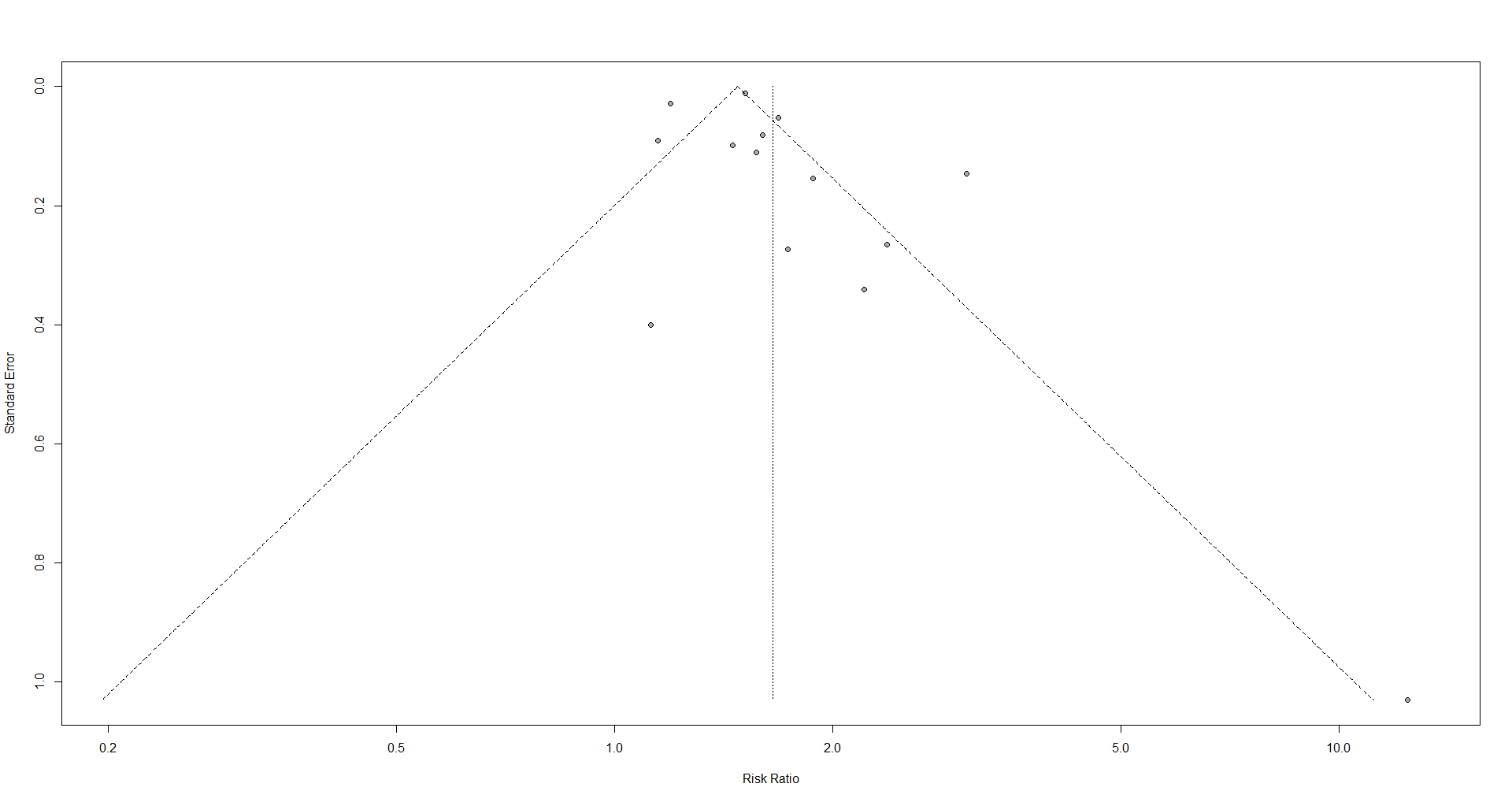
 One outlier, Graziosi et al. can be seen. Reason for this is the very low event rate (1 in late term). The weight of this study is 0.7%.

Appendix H - Sensitivity Analyses

Figure H1 Sensitivity Analysis for NICU admission


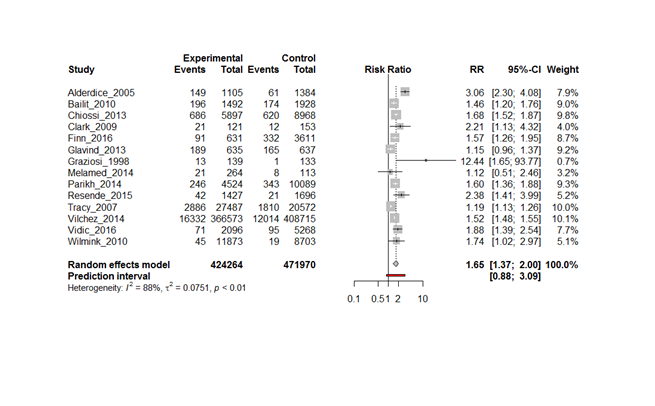

Supplement: Supplementary file 1 — Additional file 1: Appendix A. Search strategies. Appendix B. Included and Excluded Studies. Appendix C. Study characteristics of included studies. Appendix D. Risk of bias assessment with ROBINS-I. Appendix E. Results of individual studies. Appendix F. Results of individual studies (graphical illustration). Appendix G. Funnel plot for NICU admission. Appendix H. Sensitivity Analyses. [file 12884_2020_3036_MOESM1_ESM.docx]
